# Differential network analysis reveals the genome-wide landscape of estrogen receptor modulation in hormonal cancers

**DOI:** 10.1038/srep23035

**Published:** 2016-03-14

**Authors:** Tzu-Hung Hsiao, Yu-Chiao Chiu, Pei-Yin Hsu, Tzu-Pin Lu, Liang-Chuan Lai, Mong-Hsun Tsai, Tim H.-M. Huang, Eric Y. Chuang, Yidong Chen

**Affiliations:** 1 Greehey Children’s Cancer Research Institute, University of Texas Health Science Center at San Antonio, San Antonio, TX, United States of America; 2 Department of Medical Research, Taichung Veterans General Hospital, Taichung, Taiwan; 3 Graduate Institute of Biomedical Electronics and Bioinformatics, National Taiwan University, Taipei, Taiwan; 4 Department of Molecular Medicine/Institute of Biotechnology, University of Texas Health Science Center at San Antonio, San Antonio, TX, United States of America; 5 Institute of Epidemiology and Preventive Medicine, National Taiwan University, Taipei, Taiwan; 6 Graduate Institute of Physiology, National Taiwan University, Taipei, Taiwan; 7 Bioinformatics and Biostatistics Core, Center of Genomic Medicine, National Taiwan University, Taipei, Taiwan; 8 Institute of Biotechnology, National Taiwan University, Taipei, Taiwan; 9 Department of Epidemiology and Biostatistics, University of Texas Health Science Center at San Antonio, San Antonio, TX, United States of America

## Abstract

Several mutual information (MI)-based algorithms have been developed to identify dynamic gene-gene and function-function interactions governed by key modulators (genes, proteins, etc.). Due to intensive computation, however, these methods rely heavily on prior knowledge and are limited in genome-wide analysis. We present the modulated gene/gene set interaction (MAGIC) analysis to systematically identify genome-wide modulation of interaction networks. Based on a novel statistical test employing conjugate Fisher transformations of correlation coefficients, MAGIC features fast computation and adaption to variations of clinical cohorts. In simulated datasets MAGIC achieved greatly improved computation efficiency and overall superior performance than the MI-based method. We applied MAGIC to construct the estrogen receptor (ER) modulated gene and gene set (representing biological function) interaction networks in breast cancer. Several novel interaction hubs and functional interactions were discovered. ER+ dependent interaction between TGFβ and NFκB was further shown to be associated with patient survival. The findings were verified in independent datasets. Using MAGIC, we also assessed the essential roles of ER modulation in another hormonal cancer, ovarian cancer. Overall, MAGIC is a systematic framework for comprehensively identifying and constructing the modulated interaction networks in a whole-genome landscape. MATLAB implementation of MAGIC is available for academic uses at https://github.com/chiuyc/MAGIC.

Dysregulation of oncogenes is one of the main causes of cancer. Through gene mutation or copy number amplification, the continual activation of oncogenes stimulates downstream signaling transduction to drive tumor proliferation and metastasis. These oncogenes can not only perturb gene expression, but also disrupt gene interactions. For example, a recent study showed that oncogenic *KRAS* modulates HIF-1α and HIF-2α target genes and in turn regulates cancer metabolism^1^. Luo *et al.* demonstrated that *COPS3*, *CDC16*, and *EVI5* were associated with patient survival under Ras modulation^2^. Estrogen receptor (ER), the primary oncogene in the luminal type of breast cancer, was reported to coordinate coexpression of keratin genes^3^ from a dataset composed of over 100 primary breast tumors^4^. Furthermore, upon 17β-estradiol stimulation, the transcription factor (TF) regulatory network was found to be temporarily rewired in the human MCF7 breast cancer cell line^5^. These reports suggest the modulation capability of estrogen and its receptor protein (reviewed in ref. 6). Other studies also identified oncogene-modulated microRNA-gene regulation, gene-gene interaction, chemical-gene perturbation, and protein-protein interaction in cancers^7^,^8^,^9^. These studies demonstrate that oncogenes play a modulatory role in the differential interaction of both genes and molecular functions.

With advances in microarrays and next-generation sequencing technologies, gene interaction networks have been constructed to understand the gene-gene interactions in cancer^10^,^11^,^12^. Some studies have employed these networks for clinical applications such as classification and prognosis prediction in breast cancer^13^,^14^. In order to extend this work, the concept of “differential network biology” was introduced to take into account the condition-specific rewiring of genetic and protein maps (reviewed in ref. 15). *In vitro* investigations have been carried out on networks of protein-protein^16^, protein-DNA^17^,^18^, and genetic interactions^19^. The results indicated the comprehensive effects of modulation of the interactome at any given point. For analyzing differential networks, some methods employ unsupervised hierarchical clustering to identify modules of gene pairs that share common patterns of differential coexpression between conditions^20^,^21^,^22^. Although these methods provide an overview of the inner structures of differential networks, they are limited in specifically dissecting the mechanisms governed by the cellular conditions determined by the status of a modulator gene, such as ER. Alternatively, the modulation-based methods are developed to directly identify core differential networks modulated by a modulator. One class of such methods is based on the comparison of topological changes and rewiring among interaction networks each derived from a particular cellular condition^19^,^23^,^24^ (illustrated in Supplementary Fig. S1A). Since the fundamental components of these condition-specific networks are largely composed of static interactions, elucidating and analyzing the rewiring of these complex networks remain a challenging task. Another class of the modulation-based approach is to directly identify the modulated genomic interactions of which regulatory strength is significantly changed between conditions, and focuses on the network formed by these interactions only^16^ (Supplementary Fig. S1B). Several algorithms have been developed based on this approach and adopt the mutual information (MI) method to systematically explore the modulated interaction networks in cancer^7^,^25^,^26^. For example, modulator inference by network dynamics (MINDy) infers the post-translational modulation of TFs from microarray expression datasets^25^. Based on a different hypothesis, another MI-based method, namely Differential Multi-Information (DMI), was developed to infer whether a set of genes (*i.e.*, targets of a TF) are differentially correlated between conditions^27^. Specifically, DMI measures multivariate MI among genes, while MINDy computes pairwise MI between a TF and its targets. However, since these algorithms utilize computationally expensive permutation tests for statistical inference, they highly rely on *a priori* knowledge, such as TF target genes and binding sites, to reduce the amount of computation. Novel modulated interactions beyond prior knowledge remain uncharted territory.

In this study, we present a novel algorithm, modulated gene/gene set interaction (MAGIC) analysis, to systematically identify modulated interactions at two levels, the gene level and the gene set level. While genes are players in genomic regulation, gene sets represent categories of biological function and can bring comprehensive interpretation to biological observations^28^. Instead of utilizing prior knowledge to reduce the number of interactions being tested, MAGIC can efficiently examine genome-wide combinations of gene (and gene set) pairs based on the proposed statistical model. Our simulation confirmed the efficiency of MAGIC algorithm in comparison with the MI-based methods. Using breast cancer gene expression profiling datasets, we applied MAGIC to construct a modulated interaction networks by ER. By incorporating clinical survival information, the analysis further illuminated the interplay among ER, TGFβ, and NFκB, and their association with tumor progression and patient survival. The results were verified in independent breast cancer datasets. Using MAGIC, we also assessed modulated interaction networks of another hormonal cancer, ovarian cancer, and identified both cancer type-independent and type-specific features of ER modulation, further demonstrating the capability of MAGIC in elucidating ER-modulated signaling and providing better understanding of complex cancer interactomics.

## Results

### Modulated gene/gene set interaction (MAGIC) analysis

**Figure 1:**
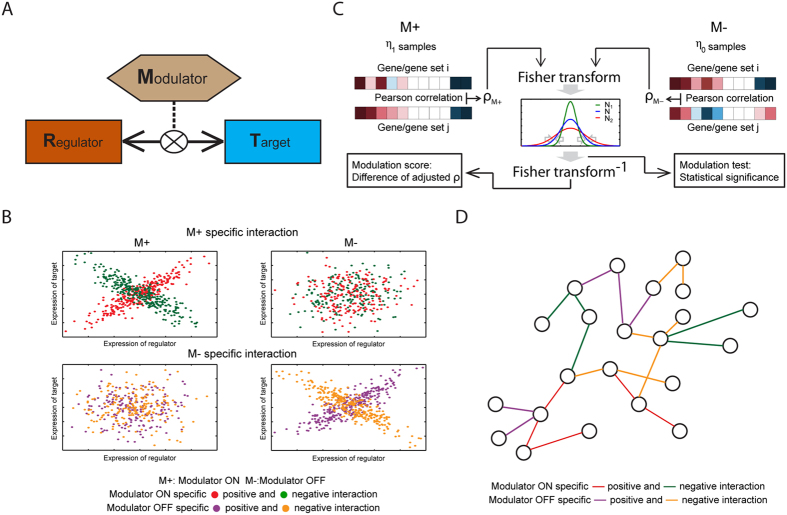
Illustration of the proposed algorithm for modulated gene/gene set interaction (MAGIC) analysis. (**A**) Illustration of modulated interaction. From the viewpoint of modulated interaction, the strength of interaction between regulator and target is dependent on the status of the modulator (indicated by M). (**B**) Examples of the modulated interaction pairs. The MAGIC method is designed to infer the interaction pairs that exhibit significantly intensified positive or negative correlation in one state of modulation (“ON” (M+) or “OFF” (M−)) compared to the other. (**C**) Schematic illustration of MAGIC. The correlation coefficients of each pair of genes (or gene sets) in M+ and M− samples are Fisher transformed and statistically tested for a difference between the M+ and M− samples. MAGIC infers modulated interaction pairs by two criteria: statistical significance of the modulation test and difference of adjusted coefficients (modulation scores). Mathematical details are described in the Methods and Supplementary Methods sections. (**D**) The modulated interaction network. The significantly modulated interaction pairs are merged and visualized in networks for dissecting the systematic view of modulated signaling. A schematic flowchart of MAGIC is shown in Supplementary Fig. S2.

MAGIC infers pairs of genes (or gene sets) whose expression levels (or enrichment scores) are correlated in a modulator-dependent manner. Fig. 1A illustrates how MAGIC can dissect the modulated functional interactions. The modulator (M) is a gene or protein that influences (either activates or suppresses) the interaction of regulator–target (R–T) pairs. The regulator and target could be genes or biological functions, where the latter are represented by gene sets and their activities are estimated by summarizing the expression of genes in gene sets. The interactions can be classified into four states as shown in Fig. 1B: positive or negative interaction specifically when the modulator is active (M+) or inactive (M−). MAGIC measures the difference in correlation coefficients between states to identify the modulated R–T pair. As shown in Fig. 1C, the Pearson correlation was applied to estimate the coexpression of gene pairs. We employed Fisher and inverse Fisher transformations on correlation coefficients to eliminate the biases arising from different sample sizes (*i.e.*, M+ and M−). The modulation score 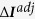, that is, the difference in the adjusted correlation, was then developed to measure the modulated interactions (Equation (9)). The statistical significance (the modulation test) was assayed on a sample-size-unbiased basis (Equation (5)). The mathematical model is detailed in the Methods section. The identified R–T pairs that met the selection criteria in terms of 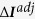 (Equations (10) and (11)) and *P*-values (Equations (6) and (7)) were constructed into networks, providing a systematic view of changes in gene and gene set interactions. The regulated network only works when specifically when the modulator is active or inactive (Fig. 1D). Analysis flowchart of MAGIC is shown in Supplementary Fig. S2. Through the proposed algorithm, a systematic study of modulator-specific interaction networks was carried out in breast and ovarian cancers.

### Performance evaluation of MAGIC and MI-based method

We utilized simulated datasets to evaluate the performance of MAGIC and the MI-based method. The simulated datasets were synthesized using three parameters: (i) sample size (*N* = 30, 100, 300, 500, and 1,000), (ii) proportion of M+ samples (75%, 50%, and 25%), and (iii) correlation coefficient in M+ samples for M-modulated pairs (*corr*_*M+*_ = 0.3, 0.7, and 1.0 (low, moderate, and high correlation), while *corr*_*M−*_ = 0). Expression levels of 5,000 gene-pairs were simulated from a bivariate normal distribution for each combination of parameter settings. The expression data were added with white noise signals and scaled (Methods). We used two simulation configurations, one with an unbalanced number of modulated gene pairs (20%) and the other with a balanced number (50%). Performance was evaluated using the measurements of precision, recall, accuracy, and computation time. We note that another class of algorithms (*i.e.*, clustering-based methods) clusters pairs of genes based on the patterns of differential coexpression instead of assessing the statistical significance of individual pairs; therefore, we did not include it in the comparison study with MAGIC (see the Discussion section).

Using the unbalanced design, MAGIC achieved overall high precision, recall, and accuracy (mean = 0.96, 0.75, and 0.95, respectively; Table 1). Low precision and recall were observed in datasets with moderate/low *corr*_*M+*_ with small sample size (*N* = 30 or 100) and/or small proportion of M+ samples (25%). Although the MI-based method achieved generally moderate precision (mean = 0.41), the recall was quite low (mean, 0.09) (Table 1), suggesting moderate false-positive and high false-negative rates. Overall, MAGIC attained considerably higher precision in 43 (95.6%) of the 45 simulation datasets and higher or equal recall in all cases than the MI-based method. In terms of accuracy, MAGIC outperformed the MI-based method by a wide margin (mean, 0.95 vs. 0.82; Table 1).

We also compared computation time between the two methods. MAGIC completed significance evaluation of 5,000 gene-pairs in 5.1 (*N* = 30 with 25% of M+ samples and moderate *corr*_*M+*_) to 8.1 (*N* = 500 with 25% of M+ samples and high *corr*_*M+*_) seconds, while the MI-based method, largely due to the permutation process, used 691 (*N* = 30 with 25% of M+ samples and moderate *corr*_*M+*_) to 3,372 (*N* = 1,000 with 75% of M+ samples and high *corr*_*M+*_) seconds (Table 1). On average, MAGIC achieved about 300-fold acceleration in computation time compared to the MI-based method.

We identified comparable trends in the simulated datasets with balanced design. The mean differences in performance between the two methods were 0.35, 0.67, and 0.33 for precision, recall, and accuracy, respectively (Supplementary Table S1). MAGIC, again, outperformed the MI-based method in computation by about 300 folds (Supplementary Table S1).

Taken together, the MI-based method suffers from high type-II errors and expensive computation, and requires large sample size to reach desirable accuracy. It is largely due to intrinsic limitations of calculating mutual information and evaluation of significance; while MAGIC, facilitated by the statistical model built on Pearson correlation, greatly improved the performance over a broad range of simulated datasets.

### The ER-modulated gene interaction network (ER-MGIN) in breast cancer

**Figure 2:**
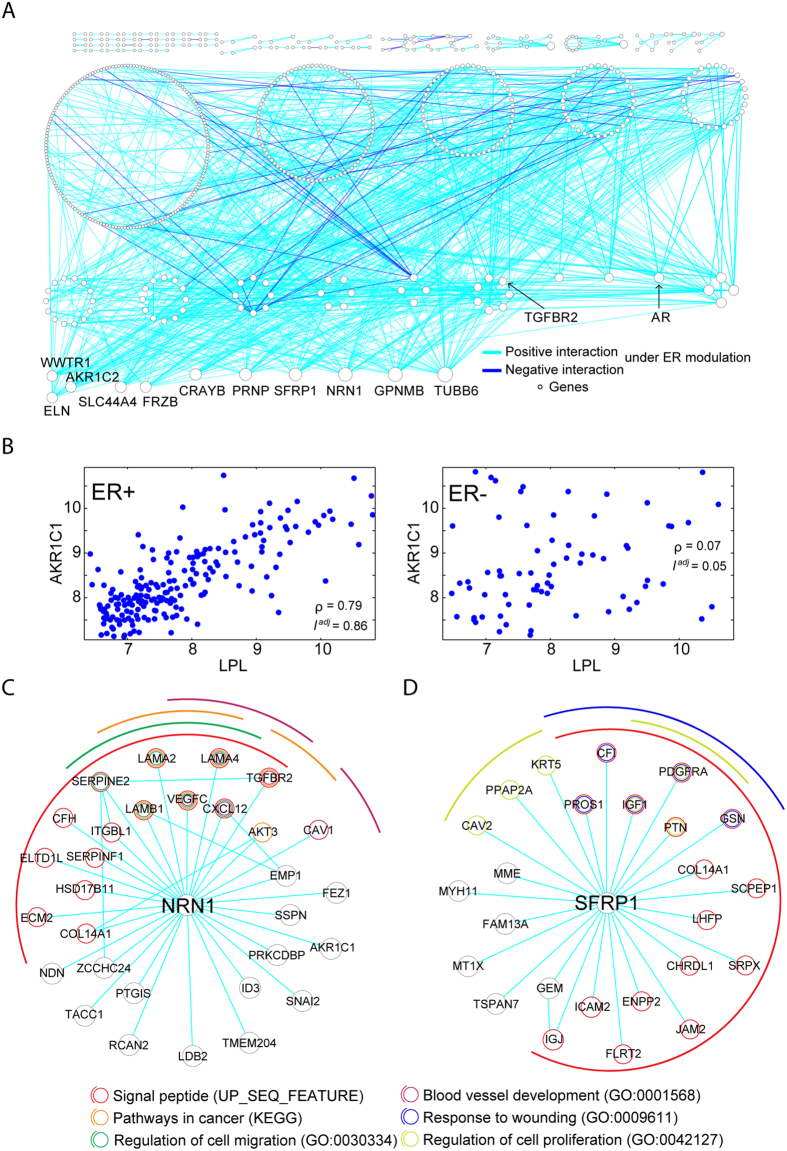
The ER modulated gene interaction network (ER-MGIN) in breast cancer. We applied MAGIC to the GSE2034 breast cancer dataset and inferred 883 significant ER-modulated gene pairs which involved 604 genes. (**A**) The ER-modulated gene interaction network. The network was constructed by merging the identified 883 gene pairs, with nodes and edges denoting genes and ER-modulated interactions, respectively. Node sizes are proportional to the degrees (number of first-order neighbors) of genes, and genes with identical degree are arranged in one circle. List and summary of ER-MRTPs are provided in Supplementary Table S3. (**B**) Scatter plots of the *AKR1C1*−*LPL* gene pair, which had the highest *∆I*^*adj*^ score of 0.81 among all ER-modulated regulatory gene pairs. Raw correlation coefficients of the two genes are 0.79 and 0.07 in ER+ and ER− samples, respectively. (**C**) Subnetwork and functional annotations of *NRN1* and its ER-modulated partners. (**D**) Subnetwork and functional annotations of *SFRP1* and its modulated partners.

Overexpression of ER is a key feature of most breast cancers. Although ER-regulated genes and functions have been widely identified, system-level gene/function modulation was uncharted territory. We applied the MAGIC algorithm to the expression profiles of breast tumors to illustrate how MAGIC resolves the modulated gene network. Summary of datasets used in the study is shown in Supplementary Table S2. Dataset GSE2034, containing expression profiles of 209 ER-expressing (ER+) and 77 non-expressing (ER−) breast tumors, was utilized to identify the M+ and M− states for ER. After excluding the non-informative genes with low signal (mean probe-set intensity <6 in log_2_ scale) or low variations (coefficient of variation <5%) across 286 samples, 5,308 informative genes were analyzed by MAGIC for identification of ER-modulated R–T pairs (ER-MRTPs). A total of 883 ER-MRTPs, including 604 genes (ER-modulated genes, ER-MGs), passed the selection criteria (Bonferroni *adj-P*-value < 0.05 and 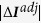 >0.6). ER-modulated gene pairs are tabulated in Supplementary Table S3A. Interestingly, all identified pairs were ER+ modulated, *i.e.*, intensified correlation was observed specifically in ER+ samples. A total of 830 out of the 883 ER-MRTPs had an ER+ specific positive correlation, while the other 53 pairs were negatively correlated. Notably, the ER-MRTPs accounted for a tiny portion (0.17%, 883 out of 527,202) of the R–T pairs formed in the ER+ samples (Bonferroni adjusted correlation *P* < 0.05 in ER+ samples). The ER-modulated gene interaction network (ER-MGIN) is showed in Fig. 2A, with nodes and edges denoting ER-MGs and ER-MRTPs. We note that ER-dependent correlation of ER-MRTPs was not necessarily attributed to differential expression of component ER-MGs between ER+ and ER− samples. Taking the ER-MG pair of *AKR1C1*–*LPL*, which had the highest 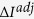 of 0.81, as an example, the correlation coefficient reached as high as 0.79 (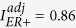) in the ER+ tumors but only 0.07 (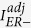 = 0.05) in the ER− tumors (Fig. 2B), while neither of the two genes exhibited significant differential expression between ER+ and ER− states (see Supplementary Fig. S3). Among the 604 ER-MGs, only 138 genes (22.9%) were differentially expressed (Bonferroni adjusted *t*-test *P* < 0.05).

The number of ER-MRTPs of each ER-MG is listed in Supplementary Table S3B. There were 11 genes involved in 20 or more ER-MRTPs. For those genes, we annotated these hub genes with gene symbols in Fig. 2A. To understand the functional enrichment of the hub genes, we applied functional annotation analysis to each of the hub genes together with their ER-modulated partners. The results showed the top 4 hub genes were all enriched in expression of the signal peptides (Fig. 2C,D and Supplementary Fig. S4). The KEGG pathway “pathway in cancer regulation of migration”, and the gene ontology terms of “regulation of cell migration” and “blood vessel development” were enriched for the partner genes of *NRN1* (Fig. 2C). Interaction partners of *SFRP1* exhibited enrichment in “response to wounding” and “regulation of cell proliferation” (Fig. 2D). We also found that the oncogene, *AR*, and the immune-related genes, *STAT3* and *TGFBR2*, were differentially regulated in the ER-modulated network. *AR* is the most crucial dysregulated oncogene in prostate cancer. The results showed that *AR* affects the functions of phosphoproteins, DNA binding, and the Golgi apparatus, at least partially, through ER modulation (Supplementary Fig. S5). The biological effects of *TGFBR2* and *STAT3* have been extensively studied in cancer and the immune system. *TGFBR2* and *STAT3* were found to be involved in the regulation of glycoproteins and acetylation under ER modulation, respectively (Supplementary Fig. S5). Collectively, these results show that our algorithm can successfully detect interaction network interactions and connect the modulated hub genes with their partner genes that affect well-studied functions.

We further sought to verify the R–T pairs in two breast cancer datasets, GSE2990 and GSE4922. Each dataset contains more than 100 samples (Supplementary Table S2). Of the 883 ER-MRTPs in the GSE2034 dataset, 59.2% and 71.7% were validated (with Bonferroni *adj*-*P* < 0.05) in GSE2990 and GSE4922, respectively. Remarkably, the top 50 ER-MRTPs achieved even higher validation rates (90.0% and 94.0%) in the two validation datasets. Overall, the 883 pairs were significantly overlapped with the results obtained from the two validation datasets (both Fisher’s exact test *P*-values ~0; hypergeometric *P* = 7.65 × 10^−32^ and 1.15 × 10^−162^). Though, the Jaccard index was quite low (0.27% and 1.22%), largely due to the very stringent criteria we set to identify the most significant ER-MRTPs and the limited sample sizes of the validation datasets. Taken together, the data demonstrated the reproducibility of results identified by MAGIC.

### The ER-modulated gene set interaction network (ER-MGSIN) in breast cancer

**Figure 3:**
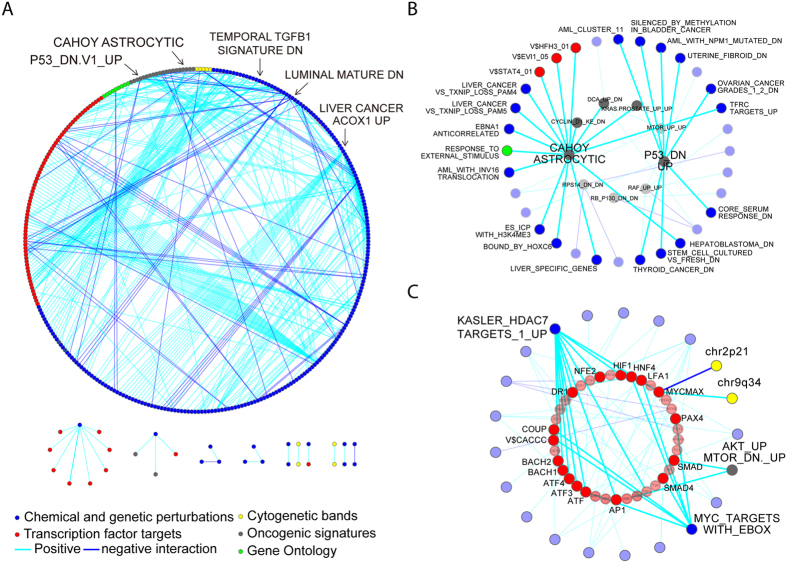
The ER modulated gene set interaction network (ER-MGSIN) in breast cancer. The MAGIC method can be also applied to identify modulated interactions among functions and pathways. (**A**) The ER-modulated functional gene set interaction network. The network was built by incorporating the 487 significant ER-modulated gene set interaction pairs composed of 604 individual gene sets. Each node and edge represent gene sets and ER-modulated interactions of pairs of gene sets, respectively. List and summary of ER-MRTPs are provided in Supplementary Table S4A,B. (**B**) Sub-network of oncogenic signature gene sets and their ER-modulated partners. (**C**) Sub-network of gene sets of TF targets and their ER-modulated partners.

To move beyond the modulated interaction network on the single-gene level, we applied MAGIC to function/pathway-level analysis based on a gene set approach. Here the activities of pathways and biological functions were modeled by enrichment scores of corresponding gene sets. After data pre-processing (detailed in the Methods and Supplementary Methods sections), a total of 2,026 gene sets underwent MAGIC for ER-modulated gene set interaction. These gene sets were defined from 5 categories: curated chemical or genetic perturbations (CGP), transcription factor targets (TFT), gene ontology terms (GO), oncogenic signatures (OS), and cytogenetic bands (CB). With identical criteria (Bonferroni *adj-P* < 0.05 and 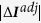 > 0.6), we identified 487 ER-MRTPs composed of 350 ER-modulated gene sets (ER-MGSs). Similar to the results of gene-level analysis, all ER-MRTPs exhibited ER+ modulated interaction. The ER-modulated gene set interaction network (ER-MGSIN) was constructed by merging the ER-MRTPs (Fig. 3A). Among the ER-MRTPs, 398 and 89 pairs showed ER+ specific positive and negative correlation, respectively. A detailed list and summary of ER-MRTPs are presented in Supplementary Table S4A,B. Of the 487 ER-MRTPs, 71.3% and 84.4% were validated in GSE2990 and GSE4922, respectively (with Bonferroni *adj*-*P* < 0.05). The validation rates of the top 50 pairs reached 88.0% in both of the validation datasets. Generally, the 487 pairs were in line with those identified from independent analyses of the two datasets (Fisher’s exact test *P* = 0.003 and ~0; hypergeometric *P* = 7.67 × 10^−3^ and 8.52 × 10^−64^), while the Jaccard index was still unsatisfactory (0.05% and 0.25%).

The connectivity of ER-MGSIN was 2.78 (the average number of ER-MRTPs directly connected to each gene set). Gene sets in the constructed network were found to be highly linked to each other, indicating that ER modulates a complex functional signaling cascade (Fig. 3A). The ER-MRTP between HUANG_DASATINIB_RESISTANCE_UP and VALK_AML_CLUSTER_11 had the highest unadjusted correlation coefficient (0.81) in ER+ samples. AMIT_EGF_RESPONSE_480_HELA and SCHLOSSER_SERUM_RESPONSE_UP were found to have the highest modulation score 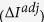 of 0.76. The gene set LEE_LIVER_CANCER_ACOX1_UP, which is composed of the up-regulated genes in mouse liver cancer after overexpression of *ACOX1*, accounted for the largest number of ER-MRTPs (35).

To dissect the complex network, we stratified it into 5 sub-networks based on the original definition of these gene sets (Fig. 3A). Through the sub-networks shown in Fig. 3B,C and Supplementary Figs S6 and S7, we interpreted that several well-studied functions/pathways were involved in the regulation of important biological functions under ER modulations. In the OS sub-network, the well-known oncogenes mTOR, cyclin D, and RAF were found to be actively regulating other gene sets in an ER+ dependent manner (Fig. 3B). The hub node CAHOY_ASTROCYTIC, which includes the up-regulated genes in astrocytes, was involved in 16 ER-MRTPs (Fig. 3B). Among the 16 ER modulated partners, three were OS gene sets, including KRAS.PROSTATE_UP.V1_UP, DCA_UP.V1_DN, and CYCLIN_D1_KE_.V1_DN. Also, CAHOY_ASTROCYTIC was found to regulate the target genes of transcription factors FOXI1 (V$HFH3_01), EVI1 (V$EVI1_05) and STAT4 (V$STAT4_01). The P53_DN.V1_UP gene set participated in 9 ER-MRTPs, including the stem cell related gene set BOQUEST_STEM_CELL_CULTURED_VS_FRESH_DN and the wound healing related gene set CHANG_CORE_SERUM_RESPONSE_DN.

The hub nodes of the TFT sub-network are shown in Fig. 3C. The gene set derived from MYC targets (BENPORATH_MYC_TARGETS_WITH_EBOX) was associated with 9 TFTs. Some of them have been proven to have important roles in cancer development. For instance, the activity of SMAD4 is highly correlated with tumor metastasis. The other hub-node, KASLER_HDAC7_TARGETS_1_UP, which is composed of genes up-regulated by expression of *HDAC7*, was connected to 12 TFT gene sets. Among them, MYCMAX and HIF1 have been well studied in cancer biology.

### The survival-associated TGFβ early-phase response gene set regulates NFκB under ER modulation in breast cancer

**Figure 4:**
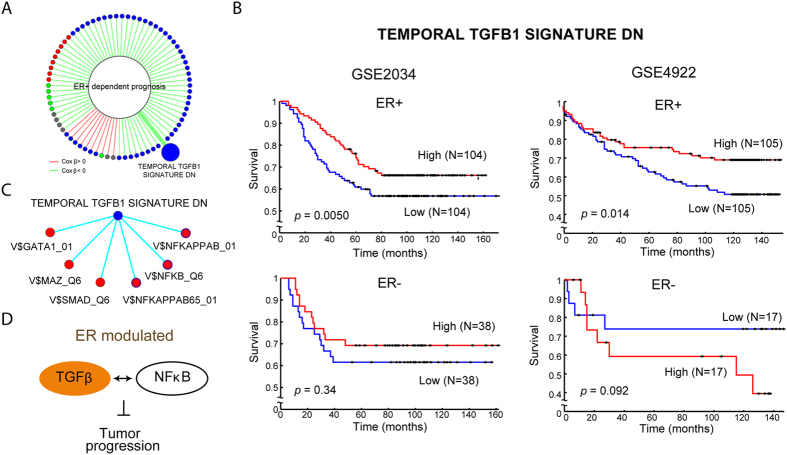
ER modulated prognostic effects of functions/pathways in breast cancer. (**A**) Visualization of the identified 75 gene set with ER-dependent survival association. (**B**) Kaplan-Meier curves of the gene set COULOUARN_TEMPORAL_TGFB1_SIGNATURE_DN in GSE2034 and GSE4922 datasets, which was originally defined as the early phase response of TGFβ. Activity of the gene set is significantly associated with patient survival, specifically in ER+ sub-cohort. Kaplan-Meier curves of GSE2990 is shown in Supplementary Fig. S9. (**C**) Sub-network of the early phase TGFβ response gene set and its ER-modulated partners. All 6 partners were TF target gene sets, including SMAD, a well-known downstream player in TGFβ signaling, and NFκB, an important regulator of inflammation and immune function. Among them, three NFκB gene sets also exhibited ER+ specific prognostic association. (**D**) Illustration of ER-modulated interaction between TGFβ and NFκB, and its effect in regulating tumor progression and patient survival.

In addition to exploring modulator-specific gene or gene set interaction, MAGIC can also incorporate clinical patient data to investigate the effects of modulation on patient survival. Among the 2,026 gene sets, we identified 610 ER-dependent prognostic gene sets, *i.e.*, a significant association between patient survival and these gene sets was specifically observed in ER+ patients (detailed in the Methods section). Seventy-five of these gene sets (23.0%) were involved in the ER-MGSIN (Fig. 4A and Supplementary Table S4C). Among them, 65 gene sets carried negative Cox beta coefficients (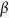), indicating ER+ specific favorable survival, and the other 10 exhibited positive *β* (adverse survival). We constructed an ER-modulated survival sub-network by extracting these prognostic genes sets and their ER-modulated partners from the ER-MGSIN (Supplementary Fig. S8 and Supplementary Table S4C). It is noteworthy that the gene set COULOUARN_TEMPORAL_TGFB1_SIGNATURE_DN, which was originally defined as group of genes overexpressed at an early phase of TGFβ^29^, exhibited ER+ dependent survival association. This gene set was reported as being associated with the molecular subtype of hepatocellular carcinoma with a less invasive phenotype^29^. Our analysis showed that in ER+ patients, high expression levels of the TGFβ gene set are indicative of better survival (Cox *P* = 4.97 × 10^−3^, Fig. 4B). However, the gene set was not significantly associated with survival in ER− patients (Cox *P* = 0.339, Fig. 4B). ER-dependent association of this gene set with patient survival was confirmed in the two validation datasets (Fig. 4B and Supplementary Fig. S9). The gene set was connected to 6 ER-MRTPs in the survival sub-network. As shown in Fig. 4C, all of the 6 gene sets were TFTs. SMAD is perhaps the best-known downstream target of TGFβ signaling. Our data revealed that ER may play a modulatory role in the interaction between TGFβ and SMAD. Also, we found that three gene sets among the NFκB targets were ER-MRTPs of the TGFβ gene set. Notably, similar to TGFβ, the three NFκB gene sets also showed ER-dependent survival associations. NFκB is an important regulator of inflammation and immune function. TGFβ is also an immune-related gene and has been reported to have dual functions in tumor biology, *i.e.*, it can act as a tumor suppressor in the premalignant state or as an oncogene during tumor progression and invasion. Our data suggest that TGFβ can interact with NFκB under ER modulation. The interaction inhibits tumor progression and in turn prolongs patient survival (illustration in Fig. 4D). The ER-modulated interaction between the TGFβ response gene set and three NFκB gene sets was confirmed in the validation datasets (see Supplementary Table S5). These observations demonstrate that ER modulation can play a crucial role in cancer prognosis and that MAGIC is capable of detecting the effects of ER-MRTPs on clinical outcomes such as survival.

### Application of MAGIC to ovarian cancer

To explore whether ER plays the role of modulator in other hormone-associated cancers, we also applied MAGIC to analyze the 185-sample primary ovarian tumor set (GSE26712). Since the status of ER was not available, we estimated ER protein expression level based on the expression level of the ER encoding gene, *ESR1*. In the gene-set analysis, among 4,256 informative genes MAGIC identified 11,411 ER+ and 173 ER− modulated gene interaction pairs, comprising a total of 1,477 ER-MGs (Supplementary Table S6A). We note that only a small, while higher than randomness, portion of these pairs were validated in an independent 420-sample dataset profiled by The Cancer Genome Atlas (TCGA) using the next-generation sequencing (observation, 597 pairs; average of 100 iterations of random sample permutation, 348.7 pairs). This reflected the potential effects of ER+/− status accuracy estimated by *ESR1* expression, lymph-node status, co-existence of other dominant modulator genes in ovarian cancer, expression measurement (log-transform or not, RPKM or TPM, etc.), and characteristics of sample population on ER modulation as well as our model (see the Supplementary Discussion section). The constructed ER-MGIN was much more complex (connectivity = 15.7, Supplementary Fig. S10) than the one in breast cancer. Among the top hub genes of the ER-MGIN we identified a potential biomarker for ovarian cancer (*PDIA3*)^30^,^31^, a BRCA1 mutation-dysregulated gene (*HNRNPA2B1*)^32^, and a gene implied to be associated with transition from normal into cancerous state in endometrial cancer, another hormone-related cancer (*DDX3X*)^33^ (Supplementary Fig. S10 and Supplementary Table S6B). Our data showed that these genes perform their functions, fully or partially, under ER modulation. We also identified several novel hub genes that were previously unreported in ovarian cancer, such as BCL2-associated transcription factor 1 (*BCLAF1*), ADP-ribosylation factor-like 8B (*ARL8B*), and SEC31 homolog A (S. cerevisiae) (*SEC31A*) (Supplementary Table S6B). We compared the ER-MGINs of breast cancer and ovarian cancer, and found that, interestingly, the two networks shared no common in hub ER-MGs (defined as nodes with connectivity in the top 5%^34^), indicating the cancer-type specific feature of ER modulation.

At the gene set level, MAGIC analyzed 2,042 pre-processed gene sets and identified 36,389 ER+ and 2,502 ER− modulated ER-MRTPs among 1,517 gene sets (Supplementary Table S6C), which again could not be satisfactorily reproduced in the TCGA dataset (observation, 2,512 pairs; average of 100 iterations of random sample permutation, 1,717.6 pairs). The constructed ER-MGSIN was highly intertwined (connectivity = 51.3, Supplementary Fig. S11). The top three hub gene sets were REGULATION_OF_IMMUNE_SYSTEM_PROCESS (GO), CADWELL_ATG16L1_TARGETS_UP (CGP), and chr4q28 (CB) (Supplementary Table S6D). The chromosome region chr4q28 was known as associated with ovarian cancer survival^35^. In the ER-MGSIN, chr4q28 interacted with a wide range of gene sets in the ER+ specific manner, such as target genes of ER, progesterone receptor (PGR), and androgen receptor (AR), response genes of sodium arsenite treatment (a compound that sensitizes ovarian cells to cisplatin^36^,^37^), gene sets characterizing ovarian cancer subtypes, and other cytobands (Supplementary Table S6E), strongly suggesting the involvement of chr4q28 in ER modulation in ovarian cancer. Among the hubs, we also identified a handful of gene sets that were known to associate essential functions, tumor growth, resistance to chemotherapeutics, or prognosis of ovarian cancer, such as immune system process, signature genes of oncogenes Src^38^,^39^,^40^ and *EZH2*^41^,^42^,^43^, and up-regulated genes in an ovarian cancer cell line upon treatment of the anticancer drug 17-AAG^44^ (Supplementary Fig. S11 and Supplementary Table S6D).

We further compared the ER-MGSINs between the two cancers and identified 2 common hub gene sets, BENPORATH_MYC_TARGETS_WITH_EBOX and CAHOY_ASTROCYTIC. BENPORATH_MYC_TARGETS_WITH_EBOX, originally defined from c-Myc target genes that contained an E-box element, was reported as associated with high-grade ER− breast tumors^45^. While the modulated partners of the c-Myc target set shared no overlap between the two cancers, a significant overlap was observed in the partners of the astrocytic gene set (Fisher’s exact test *P* = 6.68 × 10^−5^; Supplementary Fig. S12). We further examined the interaction between the TGFβ response gene set and the three NFκB target gene sets. Remarkably, a clear trend of modulation by *ESR1* was observed (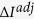 = 0.10, 0.17, and 0.12; *P* = 0.06, 0.008, and 0.03; Supplementary Table S7). However, the TGFβ response was not associated with patient overall survival in either high- or low-*ESR1* groups (data not shown). Taken together, we demonstrated the capability of MAGIC to analyze ovarian cancer and to reveal the essential role of ER modulation in ovarian cancer, similar to some extent to breast cancer, but yet distinct due to biological characteristics of ovarian cancer.

## Discussion

As we showed in our analysis, traditional gene-gene interaction analysis using gene expression profiling cannot fully capture the complicated interactions among bio-molecules in cells. The “interaction under modulation” model that investigates changes in interactions across different modulator states provides an enhanced description of the interactome. As suggested by the growing evidence that ER can function as a modulator^3^,^5^, we investigated ER-modulated interaction networks of breast cancer in this paper. To achieve our goal, a novel mathematical model, MAGIC was designed to integrate gene-level and gene set-level analyses, modulated interaction, survival analysis, and finally a simplified interaction network analysis. At the gene level, MAGIC constructed the ER-MGIN, and through analysis of key hub genes, it identified both well-studied and novel functions modulated by ER. In parallel at the gene set level, MAGIC systematically unveiled how ER modulates interactions among biological functions/pathways of CGP, GO, TFT, CB, and OS in the ER-MGSIN. MAGIC further incorporated patient survival data to pinpoint an ER-modulated interaction between TGFβ response genes and NFκB targets. The results of gene-level, gene set-level, and survival analysis were validated in two independent datasets.

By incorporating patients’ survival data, MAGIC found that the TGFβ early-phase response gene set, COULOUARN_TEMPORAL_TGFB1_SIGNATURE_DN, was associated with survival in ER-positive patients. TGFβ has two faces in breast cancer: it can play the role of tumor suppressor to inhibit epithelial cell cycle progression and promote apoptosis during early tumor growth, whereas it also acts as an oncogene that regulates the immune system and the tumor microenvironment to promote the epithelial-to-mesenchymal transition at late stages (reviewed in ref. 46). In Coulouarn *et al.*^29^, the gene set was shown to induce transcriptional activation of cell cycle arrest and apoptosis in a group of hepatocellular carcinoma patients. Different from^29^, our results showed an association between expression of the gene set and patient survival in breast cancer and in an ER-dependent manner. We speculate that the ER modulation is necessary for the early-phase TGFβ signaling to act as a tumor suppressor at an early phase of TGFβ1 treatment, thus the name “early-TGFB1 signature.” In our data, the early-TGFβ signature has 6 ER-MRTPs in the survival sub-network (Fig. 4C). One is the target gene set of SMAD, a well-known downstream signaling target of TGFβ. We also found that the ER-MRTPs of the early-TGFβ signature included three target gene sets of NFκB, an important TF that regulates immune and inflammatory responses, based on different p65 binding motifs, and all three were associated with patient survival under ER modulation. While the crosstalk between NFκB and ER has been widely studied^47^,^48^,^49^, our analysis (Fig. 4A,B) introduced a significant difference in terms of the ER dependent prognosis and interaction with TGFβ, unveiling a new potential regulation relationship: early phase of TGFβ response could co-activate with NFκB targets under ER modulation (Fig. 4C,D). This finding warrants further study. In addition to the early-TGFβ signature, we identified a total of 610 ER+ specific prognostic gene sets, and among them, 75 ER-MRTPs were further identified. Through the systematic analysis, the ER-specific crosstalk among important pathways and their prognostic effects can be delineated. For instance, a *FGFR1*-related gene set showed an interaction relationship with FOXP3 under ER modulation (Supplementary Fig. S8), yet another unexplored interaction in breast cancer discovered by our study of interaction under modulation.

MAGIC investigates ER-modulated interaction in two genomics layers, gene level and gene set level. The former reflects the mechanism by which ER directly or indirectly facilitates or suppresses gene interaction, through ER-regulated genes^50^,^51^,^52^ or its cooperating TFs, such as the forkhead DNA binding proteins^53^,^54^; while the latter enables interpretation of the “functional” effects of ER modulation. Analysis at the gene set level, representing biological pathways, cellular functions, genetic or chemical perturbation, and cytogenetic positions, has been shown to surpass single-gene methods in reproducibility, tolerance of data noise, and detection of modest changes among conditions^28^ and to greatly increase explanatory power for biological observation^55^. Our analysis supports the same observation: connectivity of a gene set in ER-MGSIN is indicative of the connectivity of genes within the gene set (Supplementary Fig. S13A, correlation = 0.149, *P* = 0.005). Furthermore, the correlation is evident between connectivity of gene sets and the proportions of hub genes in them (defined as nodes with connectivity in the top 5%^34^) (see Supplementary Fig. S13B, correlation = 0.113, *P* = 0.034). In summary, hub genes or gene sets in ER-MGSN or ER-MGSIN, respectively, likely represent two levels of precision of information flow in ER-modulated interaction networks.

**Figure 5:**
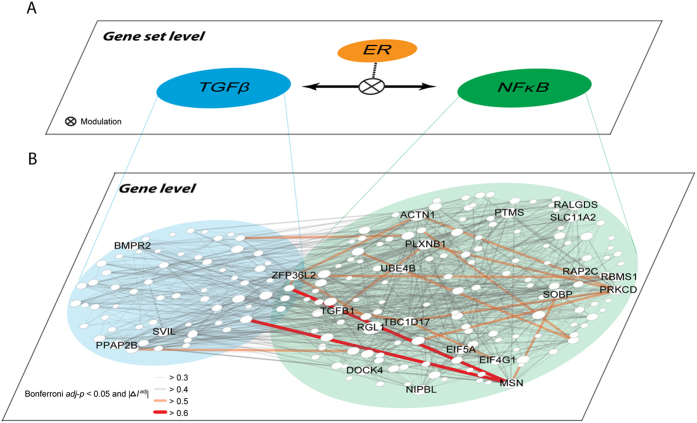
Incorporation of gene set-level and gene-level ER-modulated interaction between TGFβ and NFκB in breast cancer. (**A**) Gene set-level ER-modulated interaction between the TGFβ response gene set and the NFκB target gene set. The two gene sets, representing activities of TGFβ and NFκB proteins, were significantly correlated with each other in an ER+ dependent manner. (**B**) Gene-level ER+ dependent interaction among genes belonging to the two gene sets. Node size is proportional to the connectivity and nodes with connectivity ≥20 are labeled with gene symbols. Overrepresentation of inter-gene-set ER-MRTPs was observed (observed-to-expected ratio = 1.82).

At the gene set level, MAGIC identified significant ER-modulated interactions between TGFβ response genes and NFκB target genes in breast cancer (Fig. 5A), representing functional activities of the TGFβ and NFκB proteins, respectively. Careful examination of transcripts encoding *TGFB1*, *NFKB1*, and *NFKB2* showed modest concordant ER modulation effects at gene level (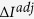 = 0.16 (raw *P* = 0.008) and 0.10 (raw *P* = 0.081), for *TGFB1*–*NFKB1* and *TGFB1*–*NFKB2*, respectively). Neither modulated interaction was significant enough to be considered in the ER-MGIN (Bonferroni *adj*-*P* = 1); however, their modulated interaction activities (represented by TGFβ and NFκB gene sets) were significant by integrating effects from their response or target genes. In addition, three interesting observations are worth noting in Fig. 5: firstly, the gene-level ER-modulated interaction network, even though constrained to only those genes in the TGFβ and NFκB gene sets, is too complex to deduce biological interpretations solely from the gene-level network; secondly, overrepresentation of inter-gene set ER-MRTPs was found between TGFβ response genes and NFκB target genes (Fig. 5B, observed-to-expected ratio = 1.82), demonstrating the stability of ER-modulated interaction of TGFβ and NFκB; and thirdly, the ER modulated gene-pairs were mostly composed of the key hub genes, such as *BMPR2*, *PPAP2B*, and *SVIL* in the TGFβ gene set and *RGL1*, *ACTN1*, and *MSN* in the NFκB target gene set (Fig. 5B). Taken together, by integrating modulated interaction analysis in two levels, MAGIC can discover critical mechanisms for maintaining ER-dependent interaction at upper levels (survival associated and ER-dependent interaction of TGFβ and NFκB gene sets) and then investigate the detailed interaction at the gene level, providing a comprehensive landscape of ER-modulated interaction.

We also applied MAGIC to explore ER modulation in ovarian cancer. Our data reveal both cancer-specific and conserved features of ER modulation. While ER-MGINs of breast cancer and ovarian cancer did not share common hub genes, at the gene set level we demonstrated that the ER modulated relationship between TGFβ and NFκB is stably maintained between breast and ovarian cancers. Indeed, the two hormonal cancers share some genomic characteristics and risk factors in common, such as mutations in *BRCA1*, *BRCA2*, *TP53*, and HER2/neu amplification^56^,^57^,^58^,^59^,^60^. However, the heterogeneity of ER+ breast cancer, the differences of mutation spectrums, and molecular profiles between the two cancers^58^,^59^ may greatly complicate the story of genomic interaction under modulation. Furthermore, it is likely that there co-exist other key modulator genes^61^, that may dilute or even overtake the effects of ER modulation. As a result, similar to other genomic features, the modulation genomic interaction appears to possess both cancer-specific and constitutive properties, partly reflecting the biological characteristics of cancer heterogeneity. Further studies that draw a pan-cancer picture of ER modulation are warranted.

The study of differential network biology agrees with biological intuition that topological changes in interaction networks are essential in cells as they are continuously receiving and responding to varied stimulation and signals. Addressing this, several algorithms have been developed for analyzing modulated or dynamic interaction, including clustering-based methods^20^,^21^,^22^ and MI-based approaches^7^,^25^,^26^. The former methods group gene pairs into modules based on their patterns of differential coexpression. The modules may comprise an overall functional landscape and reveal inner structures of the differential networks. However, it remains a challenging task to further investigate the mechanisms modulated by a modulator gene, such as ER, from the identified modules. On the other hand, MAGIC identifies statistically significant modulated gene (or gene set) pairs and incorporates clinical relevance to construct a single concise interaction network. MAGIC and the clustering-based methods fundamentally tackle different biological questions, and yield different results (a handful of modules vs. single concise network) which can hardly be compared; therefore, in the simulation study we only compared the performance between MAGIC and the MI-based methods. The MI-based approaches measure changes in probabilistic dependence between two variables. The MINDy method, one of the most well-known MI-based methods, was built to identify genome-wide post-translational modulators of TF activity by comparing CMI given the status of a modulator and MI^25^. Focusing on competing endogenous RNA regulation, Hermes is another MI-based algorithm for studying dynamic gene regulation^7^. The two algorithms conceptually infer whether acquiring knowledge of M (the modulator) improves mutual dependency of R and T (the regulator/target pair; Fig. 1A). Recently, based on MINDy, Chen *et al.* proposed the DIGGIT (driver-gene inference by genetical-genomics and information theory) method that dissected how gene mutations modulate master regulators in diseases^62^. These algorithms carried out comprehensive analyses in human cancers and other diseases, and the results were validated using biological experiments. However, the statistical inference based on random permutation tests is computationally intensive and thus limits the application in efficient genome-wide analysis. Gaussian kernel probability can be used as an estimator of entropy so that the calculation of (conditional) mutual information is estimated by covariance, which corresponds fundamentally to the correlation coefficient in the case of regulation of two genomic features^63^. MI features capability of detecting both linear and non-linear relationships between two variables. However, it was reported that gene regulation has mostly linear or monotonic relationships^64^. Therefore, correlation-based methods, such as MAGIC proposed here, can achieve equivalent or even better performance^64^, without requirement of large sample size and intensive computation, in constructing gene co-expression networks.

For identification of modulated R–T pairs, MAGIC utilizes two major criteria: the modulation scores 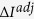 and the *P*-values of the modulation test. The former ensures biologically meaningful changes in correlation coefficients while the latter measures statistical significance. The 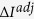 was derived from Fisher transformation of correlation coefficients followed by inverse Fisher transformation to adjust the correlation coefficients into equivalent statistical bases (*i.e.*, equal sample size). Despite the efforts to statistically eliminate the biases arisen from sample sizes, our model seemed to be still moderately affected by the sample sizes of real genomic datasets (see Supplementary Discussion), though it performed quite well in simulated datasets. This may, at least partly, arise from the fact that genomic data may not perfectly fit the normal distribution, the moderate dependency among the modulator and other genes, and the existence of other layers of genomic regulation/interactions (discussed in the Supplementary Discussion section). Notably, a critical advance in the study is that instead of the permutation strategy, the probability distribution function was derived (Equations (6) and (7)). By doing so, the statistical significance can be directly assessed without using computationally expensive permutation processes. For the analysis of ER-modulated gene interaction in this study, the critical computation processes, including calculation of pairwise correlation coefficients of 5,308 genes in 209 ER+ and 77 ER− samples, calculation of modulation test *P*-values and 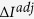 scores, and inference of ER-modulated gene pairs, were done within one minute (~51 seconds) on a machine equipped with dual quad-core (16 threads) 2.4 GHz CPUs, with less than 6 GB RAM used. However, the same processes are estimated to take about 52 days using the MI-based method (estimated based on linear computation complexity from Table 1). Overall, the advance in computation efficiency enables MAGIC to meet the challenge of genome-wide analysis of modulation networks.

The computation efficiency and flexibility makes MAGIC applicable to a variety of research topics in differential network biology. For instance, in addition to direct action of microRNAs (miRNAs) on their target genes, recent reports have unveiled an alternative role of miRNAs in facilitating crosstalk and coexpression between genes, namely the competing endogenous RNA (ceRNA) regulation^7^,^65^,^66^. The regulation strength of ceRNA is known to depend on a handful of factors, including the level of miRNAs^67^,^68^. Under this circumstance, MAGIC can be used to analyze miRNA-modulated gene-gene regulation, where the M+/− states would be high and low miRNA expression. Also, while multiple genes have been reported to be independently methylated, studies also showed that concurrent methylation of several tumor-associated genes can be associated with disease subtype^69^ and prognosis^70^ in human leukemia. In this scenario, MAGIC can be simply adapted to infer concurrent methylation of genes (with R and T representing the transformed methylation *M*-values^71^) under the modulation of disease states (M = states or subtypes) or prognosis (M = favorable/adverse prognostic factor). Furthermore, adopting the strategy of averaging correlation coefficients, Taylor *et al.* identified proteins with highly dynamic interaction with other proteins and proved that the dynamic interaction is predictive of breast cancer survival^14^. As Ideker and Krogan predicted^15^, the future analysis of “differential interaction” will become as prominent as the current analysis of “differential expression.” We expect MAGIC to be widely used in studying differential interaction and illuminating an alternative layer of modulated interaction within the complex interactome, due to MAGIC’s statistical model, flexible applicability, and computation efficiency.

## Methods

### Datasets

Gene expression microarray data of 286 lymph-node negative primary breast cancer patients (209 ER+ and 77 ER−, with NCBI/GEO^72^ accession number GSE2034^73^) who had not received adjuvant systemic treatment was used for constructing the ER-modulated gene (or gene set) interaction networks. Two additional datasets from GSE2990^74^ and GSE4922^75^ were analyzed as validation sets. For the discovery analysis of ovarian cancer, we used the GSE26712 dataset^76^. We also included the ovarian cancer dataset profiled by TCGA^58^ using the Illumina HiSeq 2000 RNA sequencing for validation purpose. Summary of datasets used in the study is shown in Supplementary Table S2. All the data were retrieved from NCBI/GEO or TCGA databases and appropriately processed to obtain gene-level expression values as described in Supplementary Methods.

### Gene sets

The gene sets of the Molecular Signatures Database (MSigDB) v3.1^77^ were analyzed in this study, including 5,982 gene sets from five categories: curated chemical or genetic perturbations (CGP), transcription factor targets (TFT), gene ontology terms (GO), oncogenic signatures (OS), and cytogenetic bands (CB). Small or oversized gene sets (containing <20 or >500 gene members) were eliminated from further analyses, except for the CB gene sets. Among the gene sets in CGP, GO, and OS, we used kappa statistics (Supplementary Equations (S1) and (S2)) to measure and cluster the gene sets with significant similarity in terms of their gene contents and assigned one centered “functionally representing gene set” for each of the clusters (details and illustration in the Supplementary Methods section and Supplementary Fig. S14). Subsequent gene set analysis was conducted using the representing gene sets.

### Gene set enrichment scoring

We proposed a gene set enrichment score to represent activities of gene sets in gene set analysis. For a given gene expression dataset, we first performed *z*-transformation: 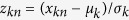, where *x*_*kn*_ is the log_2_-transfromed and normalized gene expression data of the *k*-th gene (*k* = 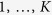) in the *n*-th sample (*n* = 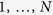), and 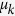 and 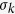 are the mean and standard deviation for the *k*-th gene, respectively. 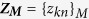 is the matrix format of *z*-values in samples with modulator status *M*, where 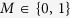 and for example, 0 representing ER−, and 1 for ER+. We defined the gene set content matrix ***G*** (number of gene sets *S* by number of genes *K*) as 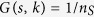, where *n*_*S*_ denotes the number of genes in gene set *s*, if *s* includes gene *k*, otherwise, 0. We calculated the gene set *enrichment score* for the *n*-th sample in the *s*-th gene set, 
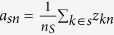
, or in the matrix format,


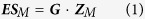


for *M* = 0 and 1. By calculating the inner product of the component of matrices ***G*** and ***Z***, we simply average all the *z*-scores of genes in a gene set for one sample. Each entry of matrix 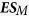 represents the degree of activation for the corresponding gene set in a sample under one modulator state.

Assuming that the gene set scores followed the standard normal distribution by the law of large numbers, we employed the statistically reliable *L*_0.05_ criterion^78^, which is ±1.96 times the standard deviation of enrichment scores in 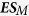 obtained from random expression levels, as the informativeness measure (*i.e.* unlikeliness to be contributed from random events) for gene sets. Gene sets with enrichment scores falling within the ±*L*_0.05_ boundary in more than 80% of the samples in either the ER+ or ER− cohort were denoted as non-informative and filtered out from subsequent analyses.

### Modulated gene/gene set interaction (MAGIC) analysis

MAGIC is designed to examine the association of two genes/gene sets whose regulatory interactions are modulated by the modulator status, *i.e.*, 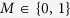 (ER− and ER+ in this study), representing the “ON” and “OFF” states of a modulator (Fig. 1A). A schematic flowchart of MAGIC is shown in Supplementary Fig. S2. Taking gene analysis as an example, we started by calculating the Pearson correlation as the measure of “interaction” between genes *i* and *j*:


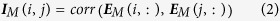


where 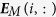 and 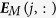 are the vectors of expression values of gene *i* and *j*, respectively, under a given modulator status 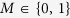. For the gene set analysis, the enrichment scores (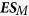 in Equation (1)) of gene sets were used in place of the gene expression abundances 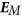.

We hypothesized that for a modulator to be relevant to a given biological system, it must exert a strong influence on a network of genes when it is functional (whether *M* = 1 or 0), but have relative weak effect otherwise. We wanted to identify a pair of genes that show a significant difference in their interaction (modulated by *M*) by statistically testing


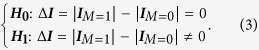


Realizing that sample correlation coefficients are prone to be biased by different sample sizes of 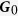 and 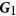, we performed Fisher transformation^79^ to project the correlation coefficients to a sample-size-free domain (criterion 1: *modulation test*), followed by inverse Fisher transformation to adjust the correlation coefficients to an assigned sample size so that equivalent sample size can be achieved for two groups of samples (criterion 2: *modulation score*). Mathematical details of the two filtering criteria are provided below.

Given the correlation coefficient 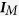 and sample size 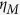, Fisher transformation, as defined in Equation (4), projects 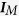 to the standard normal distribution and yields 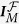.


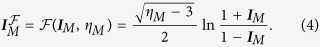


Assuming that 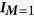 is independent of 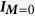, the modulation test estimates the statistical significance of


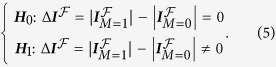


Based on the normal distribution of 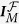, we derived the probability density function (PDF) of 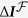 as


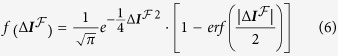


and the cumulative distribution function (CDF) as


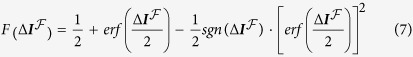


where 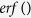 is the Gauss error function and 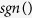 is the sign function which gives 1 or −1 for positive or negative inputs, respectively. Since the PDF and CDF were determined, the significance of 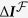 (the *modulation test P*-value) can be directly assessed. The threshold of statistical significance was adjusted for multiple testing with a Bonferroni correction.

We then performed an inverse Fisher transformation on 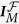 to adjust the coefficient to an assigned sample size 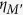. The inverse Fisher transformation followed


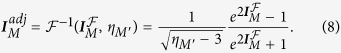


Based on the conjugate Fisher- inverse Fisher transformation, correlation coefficients sampled from two populations can be compared with the adjusted equivalent sample size of 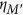. We define the *modulation score* as


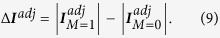


The modulator “ON” interaction can be identified as an element of 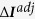 that is greater than the threshold 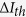



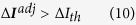


and the modulator “OFF” interaction can be identified as an element of 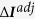 less than 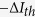



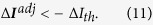


### Survival analysis

We integrated patient survival information into MAGIC. A Cox proportional hazards regression model was used to identify modulator-dependent prognostic gene sets. A gene set was defined as a modulator-dependent prognostic gene set if (i) *P*_M = 1_ < 0.01, *P*_M = 0_ > 0.1, or (ii) *P*_M = 0_ < 0.01, *P*_M = 1_ > 0.1, where *P*_M_ is the *P*-value yielded from the Cox model. The criteria were designed to catch the gene sets for which a survival association was observed specifically in one state of modulation.

### Visualization of interaction networks

The identified ER-modulated gene (and gene set) pairs were combined into networks with nodes representing genes (gene sets) and edges denoting the modulated interaction. We employed open source software Cytoscape^80^ for visualizing constructed interaction networks. For fully representing the identified information regarding genes and their interaction relationship, node and edge colors were designed to illustrate the survival association and interaction types, respectively. The complexity of regulatory relationships and signal transduction were measured by the “connectivity” (*i.e.* average number of nodes adjacent to each node) in the built network.

### Implementation of MI-based method

MI-based methods for identifying modulated interaction are typically constructed based on comparison between conditional MI (CMI) given the status of a modulator *M* and MI^7^,^25^, *i.e.*,


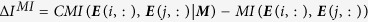


Pairs of genes exhibiting significant positive 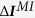 are considered as *M*-modulated regulatory pairs. In the simulation study, we calculated MI and CMI by using the MATLAB tool “MIToolbox for C and MATLAB”^81^. Statistical significance of 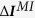 was assessed by a 1,000-time permutation test with respect to modulator status for each gene pair.

### Simulation study

We synthesized a total of 45 simulated datasets, each dataset was constructed using a specific sample size (*N* = 30, 100, 300, 500, or 1,000). For each dataset, samples were partitioned into two groups under different modulator statuses (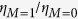 = 3, 1, or 1/3), with a particular correlation coefficient for modulated gene pairs in *M* = 1 samples (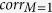 = 0.3, 0.7, or 1.0) and *M* = 0 samples (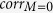 = 0). The correlation coefficient of unmodulated gene pairs was set as zero. In each dataset, expression levels of 5,000 pairs of genes were independently simulated by sampling the bivariate standard normal distribution with zero mean and a standard deviation of 10. A Gaussian white noise with 20% power of the original expression data was added. We used two main simulation configuration:

- The percentage of modulated gene pairs was set to 20% (1,000 and 4,000 hypothetical modulated and unmodulated gene pairs, respectively) in each dataset (hereafter referred to as an “unbalanced” design). All of the modulated gene pairs carried pairwise positive correlation in samples with *M* = 1 while zero correlation in samples with *M* = 0.
- The percentage of modulated gene pairs was set to 50% (2,500 and 2,500 hypothetical modulated and unmodulated gene pairs, respectively) in each dataset (a “balanced” design).

For each of the two configuration, 45 simulated datasets (5 sample sizes, 3 modulation partitions, and 3 gene-pair correlation coefficients) were generated and tested for performance using the MAGIC method and the MI-based method. Gene pairs with *P*-values < 0.001 from the modulation test (MAGIC) or permutation test (MI-based method) were called as *M*-modulated pairs. The performance was measured based on 4 measurements: precision, recall, accuracy, and computation time. Mathematical expressions of the measurements are:


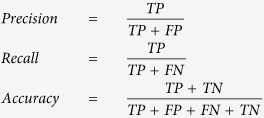


where *TP*, *FP*, *FN*, and *TN* denote the numbers of true positives, false positives, false negatives, and true negatives, respectively. The simulation study was performed and timed on a computing machine equipped with dual quad-core (16 threads) 2.4 GHz CPUs.

## Additional Information

**How to cite this article**: Hsiao, T.-H. *et al.* Differential network analysis reveals the genome-wide landscape of estrogen receptor modulation in hormonal cancers. *Sci. Rep.* **6**, 23035; doi: 10.1038/srep23035 (2016).

## Supplementary Material

Supplementary Information

Supplementary Table S3

Supplementary Table S4

Supplementary Table S6

## Figures and Tables

**Table 1:** Performance of MAGIC in comparison with MI-based methods (unbalanced design).

Measurement	Method	ρ_M+_^a^	N = 30			N = 100			N = 300			N = 500			N = 1000			Mean
		3:1^b^	1:1^b^	1:3^b^	3:1^b^	1:1^b^	1:3^b^	3:1^b^	1:1^b^	1:3^b^	3:1^b^	1:1^b^	1:3^b^	3:1^b^	1:1^b^	1:3^b^		
Precision	MAGIC	**0.3**	**0.96**	0.72	0.25	**0.98**	**0.91**	0.71	**0.99**	**0.99**	**0.97**	**1.00**	**1.00**	**0.99**	**1.00**	**1.00**	**1.00**	0.90
		**0.7**	**0.99**	**0.98**	**0.84**	**1.00**	**0.99**	**0.99**	**1.00**	**0.99**	**1.00**	**1.00**	**1.00**	**1.00**	**1.00**	**0.99**	**0.99**	0.98
		**1.0**	**0.99**	**1.00**	**0.99**	**1.00**	**1.00**	**0.99**	**1.00**	**1.00**	**1.00**	**1.00**	**1.00**	**1.00**	**1.00**	**1.00**	**1.00**	1.00
	MI	**0.3**	0.00	0.11	0.11	0.20	0.17	0.00	0.50	0.40	0.40	0.00	0.14	0.00	0.43	0.00	–	0.18
		**0.7**	0.33	0.30	0.23	0.00	0.33	0.00	**0.88**	0.25	0.00	0.64	0.43	0.33	**0.97**	0.79	0.43	0.39
		**1.0**	0.43	0.27	0.00	0.60	0.29	0.40	**0.96**	**0.93**	0.43	**1.00**	**0.99**	0.45	**1.00**	**0.99**	**0.91**	0.64
Recall	MAGIC	**0.3**	0.03	0.01	0.00	0.18	0.08	0.02	0.76	0.50	0.18	**0.96**	**0.82**	0.38	**1.00**	**0.99**	0.78	0.45
		**0.7**	0.53	0.26	0.07	**1.00**	**0.97**	0.59	**1.00**	**1.00**	**0.99**	**1.00**	**1.00**	**1.00**	**1.00**	**1.00**	**1.00**	0.83
		**1.0**	**1.00**	**1.00**	**0.84**	**1.00**	**1.00**	**1.00**	**1.00**	**1.00**	**1.00**	**1.00**	**1.00**	**1.00**	**1.00**	**1.00**	**1.00**	0.99
	MI	**0.3**	0.00	0.00	0.00	0.00	0.00	0.00	0.00	0.00	0.00	0.00	0.00	0.00	0.00	0.00	0.00	0.00
		**0.7**	0.00	0.00	0.00	0.00	0.00	0.00	0.01	0.00	0.00	0.01	0.01	0.00	0.09	0.02	0.00	0.01
		**1.0**	0.00	0.00	0.00	0.00	0.00	0.00	0.22	0.05	0.00	**0.90**	0.41	0.01	**1.00**	**1.00**	0.07	0.24
Accuracy	MAGIC	**0.3**	**0.80**	**0.80**	0.80	**0.83**	**0.81**	**0.80**	**0.95**	**0.90**	**0.83**	**0.99**	**0.96**	**0.87**	**1.00**	**1.00**	**0.96**	0.89
		**0.7**	**0.90**	**0.85**	**0.81**	**1.00**	**0.99**	**0.92**	**1.00**	**1.00**	**1.00**	**1.00**	**1.00**	**1.00**	**1.00**	**1.00**	**1.00**	0.96
		**1.0**	**1.00**	**1.00**	**0.97**	**1.00**	**1.00**	**1.00**	**1.00**	**1.00**	**1.00**	**1.00**	**1.00**	**1.00**	**1.00**	**1.00**	**1.00**	1.00
	MI	**0.3**	0.80	0.80	0.80	0.80	0.80	0.80	0.80	0.80	0.80	0.80	0.80	0.80	0.80	0.80	0.80	0.80
		**0.7**	0.80	0.80	0.80	0.80	0.80	0.80	**0.80**	0.80	0.80	**0.80**	0.80	0.80	**0.82**	**0.80**	0.80	0.80
		**1.0**	0.80	0.80	0.80	**0.80**	0.80	0.80	**0.84**	**0.81**	0.80	**0.98**	**0.88**	0.80	**1.00**	**1.00**	**0.81**	0.85
Time (sec.)	MAGIC	**0.3**	5.2	5.1	5.1	5.2	5.1	5.2	5.2	5.3	5.3	5.3	5.3	5.3	5.4	5.4	5.4	5.3
		**0.7**	5.1	5.1	5.1	5.2	5.2	5.2	5.2	5.3	5.3	5.3	5.3	5.3	5.4	5.4	5.5	5.3
		**1.0**	5.2	5.1	5.1	5.2	5.2	5.2	5.2	5.3	5.3	5.4	7.2	8.1	6.4	6.2	5.5	5.7
	MI	**0.3**	736	717	693	1020	1012	943	1579	1561	1421	2020	1960	1828	3042	2903	2681	1608
		**0.7**	726	711	691	1008	1007	950	1597	1532	1427	2041	1954	1812	3030	2905	2679	1605
		**1.0**	728	716	696	1018	1007	946	1579	1524	1454	2341	2499	2436	3372	2904	2674	1726

Measurement numbers greater than 0.80 are labeled in bold.

^a^Correlation coefficient in M+ samples for M-modulated pairs.

^b^Ratio between numbers of M+ and M− samples.
