# Supplementary material for: Differential network analysis reveals the genome-wide landscape of estrogen receptor modulation in hormonal cancers
Source: Sci Rep. 2016 Mar 14;6:23035. doi: 10.1038/srep23035 (PMC4789788; doi:10.1038/srep23035)
Supplement: Supplementary Information [file srep23035-s1.pdf]

Supplementary Information: Supplementary Methods, Supplementary Discussion, Supplementary References, Supplementary Figures S1-S14, and Supplementary Tables S1, S2, S5, and S7

Differential network analysis reveals the genome-wide landscape of estrogen receptor modulation in hormonal cancers

Tzu-Hung Hsiao*, Yu-Chiao Chiu*, Pei-Yin Hsu, Tzu-Pin Lu, Liang-Chuan Lai, Mong-Hsun Tsai, Tim H.-M. Huang, Eric Y. Chuang§, and Yidong Chen§

Supplementary Methods

Preprocessing high-throughput genomic datasets

Three breast cancer datasets, all profiled with Affymetrix Human Genome U133A Arrays (GPL96), were retrieved from NCBI Gene Expression Omnibus database (Supplementary Table S1). For each of the datasets, we employed the Robust Multi-array Average (RMA) procedures on Affymetrix .CEL files for background adjustment, quantile normalization, and calculation of probe-set-level expression values. Values were represented in $\log_2$ scale. For gene-level analysis, in the discovery dataset of breast cancer (GSE2034) a gene with multiple probes was represented by the probe carrying the largest coefficient of variation (CV). Genes with mean intensity < 6 or CV < 0.05 were considered as non-informative genes and eliminated from subsequent MAGIC analysis. Corresponding probes were selected from GSE2990 and GSE4922, respectively, for validation analysis. The ovarian cancer dataset (GSE26712) was preprocessed identically as GSE2034. For the TCGA ovarian dataset, we used the preprocessed level-3 RPKM values of 420 ovarian tumors profiled by Illumina HiSeq 2000 RNA sequencing.

Representation of similar gene sets based on kappa statistics

Despite that MSigDB collects a large volume of gene sets from independent resources, some gene sets may share highly overlapped gene contents, for a biological function may be annotated as multiple gene sets in different categories (examples in Supplementary Fig. S2A-B). Dependence between gene sets can cause bias in statistical tests and interpretation of analytical results. Addressing this, we employed kappa statistics to identify clusters of similar gene sets and find a representative gene set for each cluster. The kappa statistic characterizes the "degree of agreement" of two gene sets, considering both commonly shared (*i.e.* genes appearing in both of gene sets) and exclusive (*i.e.* genes included in neither of two sets) components of two sets of genes. Defined as

$$\kappa = \frac{p_o - p_c}{1 - p_c} \quad \text{(S1)}$$

, where p_o and p_c denote the observed and the expected proportions of genes in which two gene sets agree, respectively, the kappa statistic falls in the range of $[-1, +1]$, with large value indicating high degree of agreement. κ is approximately normally distributed (Cohen, 1960) with the standard error of:

$$\sigma_\kappa = \sqrt{\frac{p_o(1-p_o)}{N(1-p_c)^2}} \quad \text{(S2)}$$

, and the statistical significance can be tested against the null hypothesis of $p_o = p_c$. Based on the kappa statistics, functionally defined gene sets (CGP, GO, and OS) with pairwise significant agreement (Bonferroni adjusted P-values < 0.05) were defined as similar gene sets and pooled into a gene set cluster. Histogram of size of gene set clusters is shown in Supplementary Fig. S2C. For each cluster, gene set with the highest intra-cluster mean kappa statistic was selected as the representing gene set. For a gene set clusters with multiple representing gene set definitions, to conserve the most information as possible the representing gene sets with the largest sizes of gene contents was selected (examples in Supplementary Fig. S2B). Subsequent gene-set level analysis of MAGIC was conducted based on the representing gene sets.

Supplementary Discussion

MAGIC is developed based on two criteria: the modulation score ΔI^{adj} and the significance assessed by the modulation test. Specifically, we adopted the conjugate Fisher - inverse Fisher transformation to handle the biases on sample correlation coefficients caused by sample sizes. Such transformation was shown to be statistically effective (Fisher, 1915) and performed well in our simulation datasets (the Results section of main text). To test its performance in real genomic dataset, we applied MAGIC to sub-datasets generated by down-sampling the GSE2034 breast cancer data set (209 ER+ and 77 ER− samples). In the analysis of equally-sized ER+ and ER− sub-cohorts (N = 77 in each group), we identified a moderate prevalence of ER+ specific gene interaction pairs compared to ER− specific ones (average, 1,917.6 vs. 1,764.8 pairs, from ten independent down-sampling iterations). However, in the scenario generated by reversing the ER+/ER− ratio (*i.e.*, 28 ER+ and 77 ER−), a majority of significant ER-MRTPs were ER− specific (average, 42.8 vs. 6,073.3 pairs). Indeed, correlation obtained from a population with large sample size is intrinsically of high statistical power. Furthermore, the assumptions of MAGIC that high-throughput genomic data follow a standard normal distribution and the expression of modulator gene is independent of other genes may not always hold true in highly heterogeneous and complex cancer genomics. The co-existence of other key modulator genes may also complicate the problem.

On the other hand, while our findings of breast cancer (GSE2034) were validated by two independent cohorts, we failed to verify those of ovarian cancer (GSE26712) in the TCGA dataset profiled by a sequencing platform. This illuminated the possibility that while ER serves as a dominant modulator gene in breast cancer, in ovarian cancer there may coexist other key players of genomic modulation. As we discussed in the main text, changes in the mutational spectrum and molecular profiles between the two cancers could also affect the dominance of ER modulation. Furthermore, the validation rate may be influenced by the differences in clinical characteristics of the two ovarian cancer cohorts. Since in both datasets the immunohistochemical status of ER was not available, we adopted the expression level of *ESR1* as an estimation; the estimation accuracy may be limited. Also, for expression measurement and processing is diverse in the analysis of next-generation sequencing data, *e.g.*, log-transform or not, RPKM or TPM, etc., further

modifications may be made to MAGIC to carry out statistically and biologically more meaningful analyses from sequencing data. Future study that addresses these limitation is warranted.

Supplementary References

Bonome, T., *et al.* A gene signature predicting for survival in suboptimally debulked patients with ovarian cancer. *Cancer research* 2008;68(13):5478-5486.

Cancer Genome Atlas Research, N. Integrated genomic analyses of ovarian carcinoma. *Nature* 2011;474(7353):609-615.

Cohen, J. A Coefficient of Agreement for Nominal Scales. *Educational and Psychological Measurement* 1960;20(1):37-46.

Fisher, R.A. Frequency distribution of the values of the correlation coefficient in samples from an indefinitely large population. *Biometrika* 1915:507-521.

Ivshina, A.V., *et al.* Genetic reclassification of histologic grade delineates new clinical subtypes of breast cancer. *Cancer Res* 2006;66(21):10292-10301.

Sotiriou, C., *et al.* Gene expression profiling in breast cancer: understanding the molecular basis of histologic grade to improve prognosis. *Journal of the National Cancer Institute* 2006;98(4):262-272.

Wang, Y., *et al.* Gene-expression profiles to predict distant metastasis of lymph-node-negative primary breast cancer. *Lancet* 2005;365(9460):671-679.

Supplementary Figures

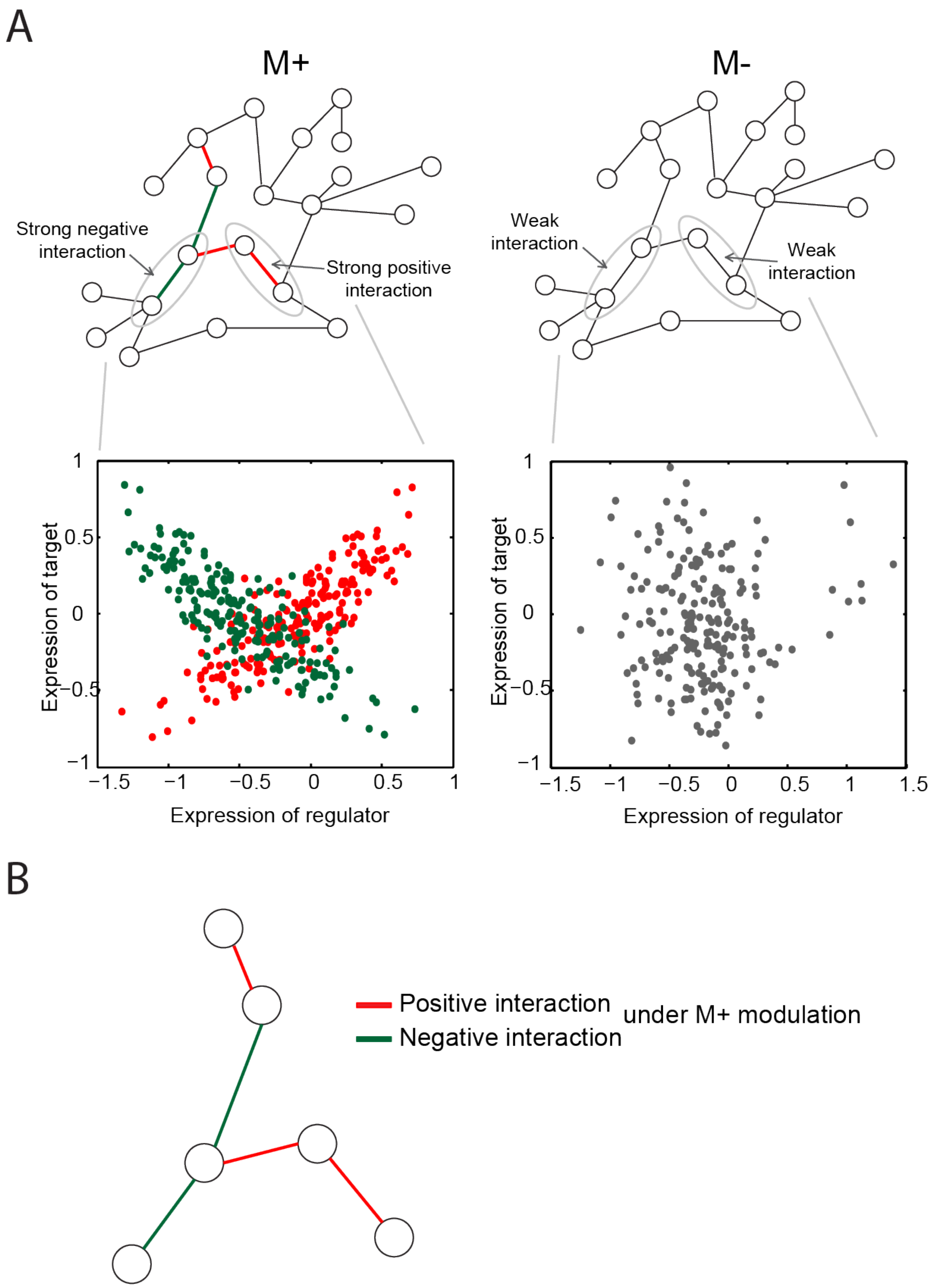

Supplementary Figure S1: Illustration of two approaches for analyzing differential interaction networks. (A) Differential networks can be analyzed by comparing interaction networks each obtained from a specific cellular condition, in terms of topological changes and rewiring. **(B)** An alternative method is to construct a constrained differential interaction network by merging the modulated genomic pairings of which regulatory strength is significantly modulated by cellular conditions.

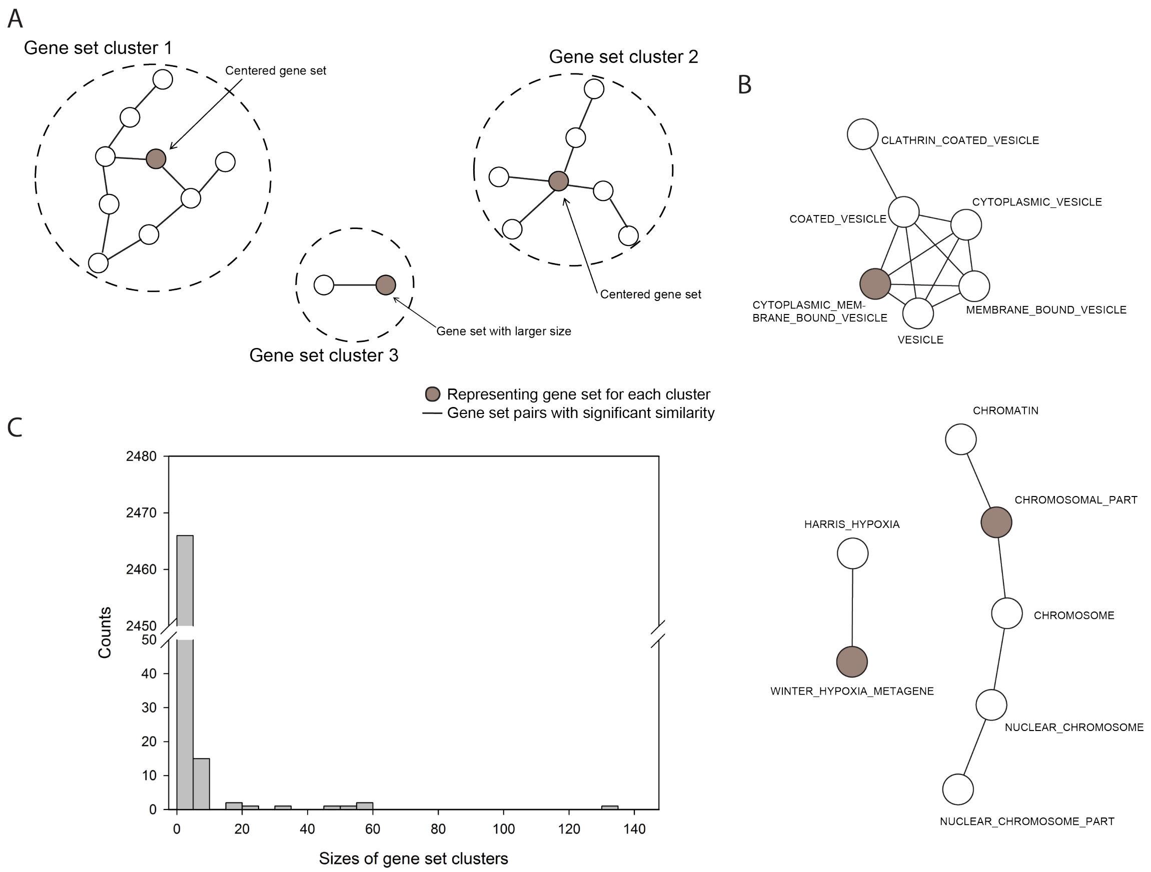

Supplementary Figure S2: Identification and representation of similar gene sets based on kappa statistics. (A) Illustration of gene set clusters and selection of representative gene sets. Kappa statistic was employed for identifying gene set clusters. Functional gene sets with pairwise significant kappa *p*-values were considered as similar and then clustered. For each gene set cluster, the gene set with the highest intra-cluster mean kappa statistic was selected as the representative gene set (illustrated as the centered gene set). For gene set cluster where two centered gene sets exist, the larger one was selected. **(B)** Real examples of identified gene set clusters. **(C)** Histogram of number of gene sets within each cluster.

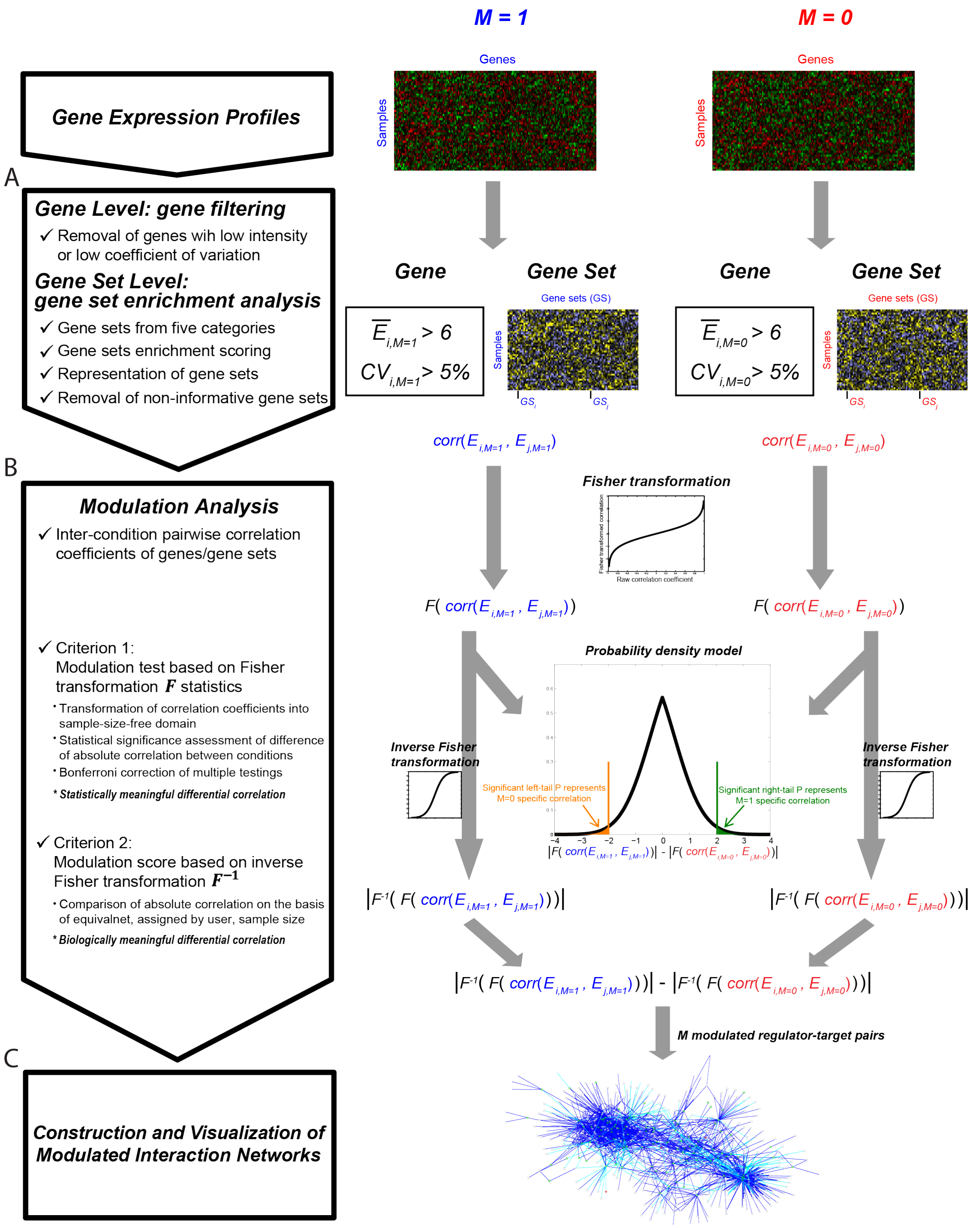

Supplementary Figure S3: Analysis flowchart of MAGIC. MAGIC is composed of three major components: **(A)** gene/gene set scoring and filtering, **(B)** modulated analysis, including two major criteria based on conjugate Fisher and inverse Fisher transformation, and **(C)** construction and visualization of the modulated interaction networks. Mathematical details are provided in the Methods section of main text.

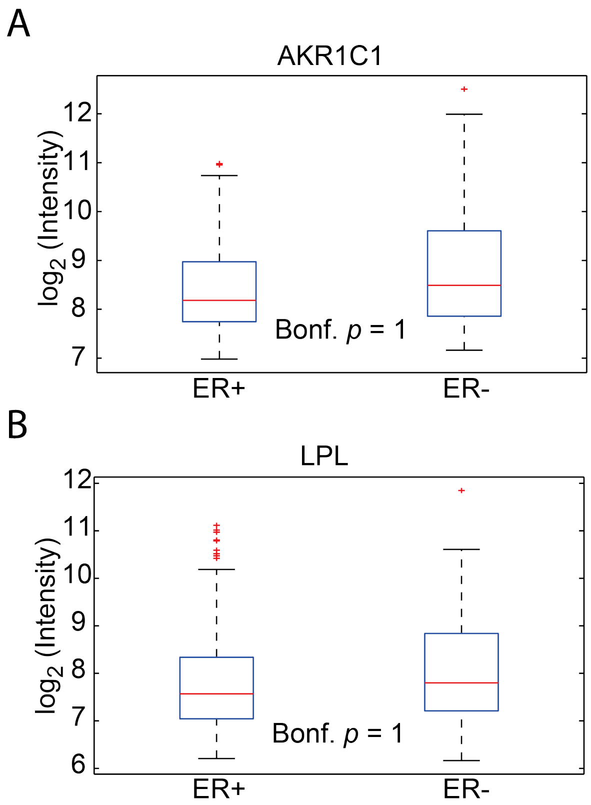

Supplementary Figure S4: Expression abundance of *AKR1C1* and *LPL* in breast cancer. *AKR1C1-LPL* gene pair had the highest modulation score among all the ER-MRTPs in breast cancer. However, neither of the two genes was differentially expressed between the states of ER. Bonferroni adjusted *t*-test *P*-values are labeled in the figure. **(A)** Box plots of *AKR1C1*. **(B)** Box plots of *LPL*.

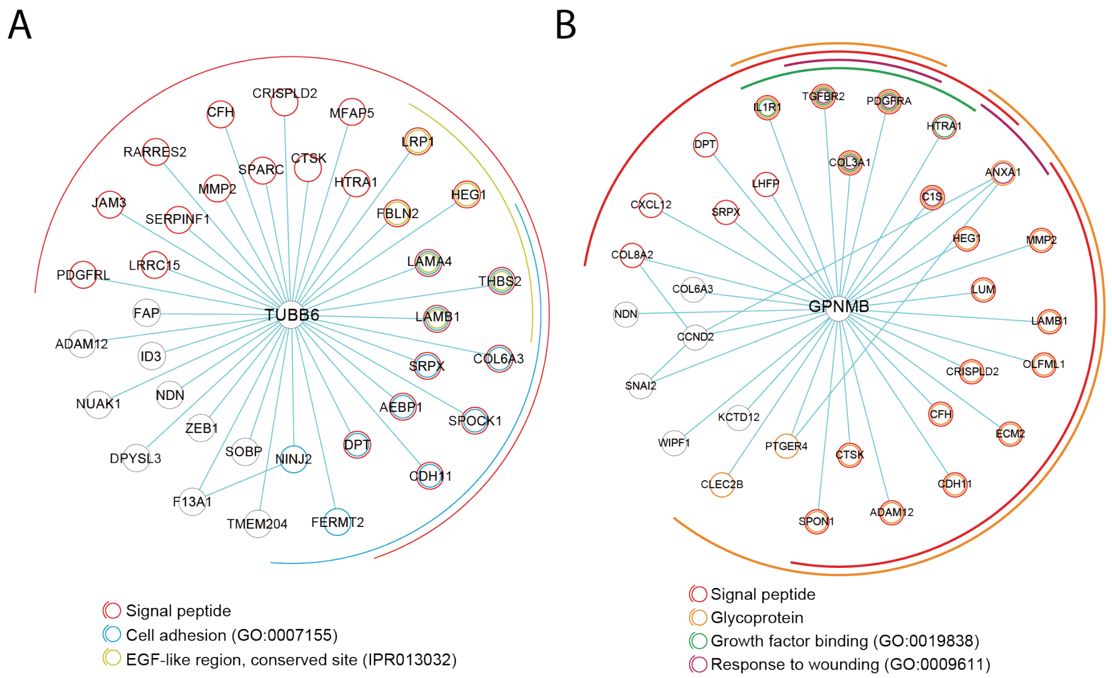

Supplementary Figure S5: The ER modulated interaction sub-networks of *TUBB6* and *GPNMB* from the ER-MGIN in breast cancer (Fig. 2A of main text). (A) Thirty-six genes were correlated with the expression of *TUBB6* under ER modulation. These genes showed enrichment functions of signal peptide, cell adhesion, and EGF-like region, conserved site. **(B)** *GPNMB* was connected to 32 genes, enriched in functions of signal peptide glycoprotein, growth factor binding, and response to wounding.

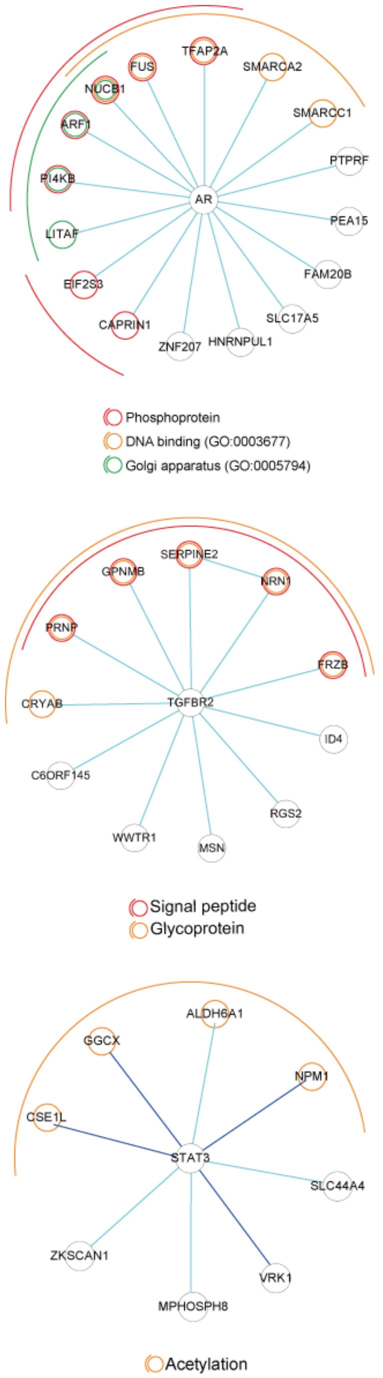

Supplementary Figure S6: The ER modulated interaction sub-networks of *AR, TGFBR2* and *STAT3* from the ER-MGIN in breast cancer (Fig. 2A of main text). *AR, TGFBR2* and *STAT3* were found to be involved in 16, 11, and 8 ER-MRTPs, respectively. The ER-modulated partners of *AR* exhibited enrichment in functions of phosphoprotein, DNA binding, and Golgi apparatus. The partners of *TGFBR2* were enriched in signal peptide and glycoprotein. Acetylation was enriched in the *STAT3* sub-network.

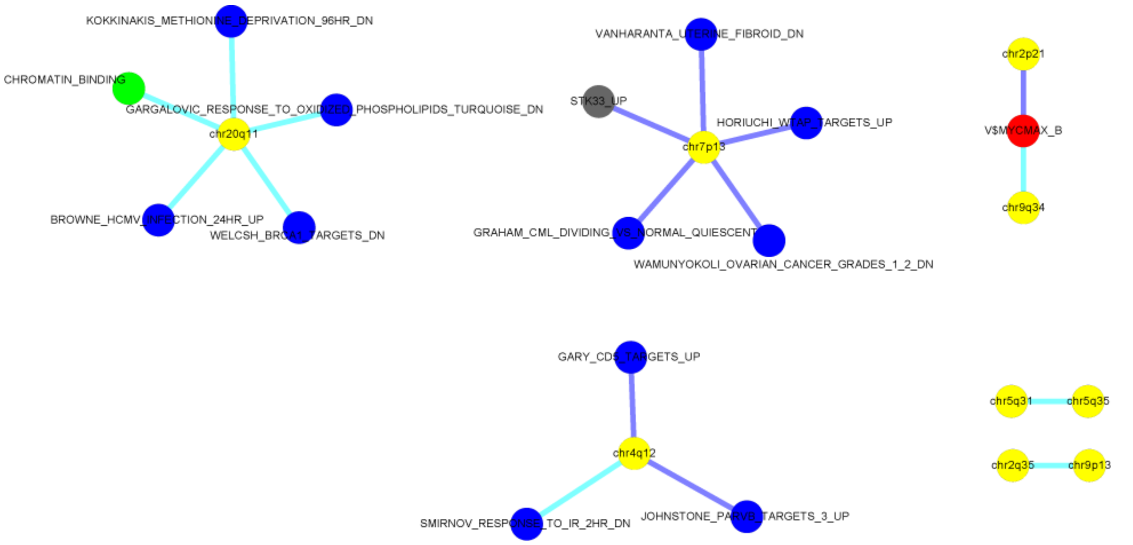

Supplementary Figure S7: The sub-network of cytogenetic bands in breast cancer. Extraction of CBs and their ER-modulated partners from the ER-MGSIN (Fig. 3A of main text).

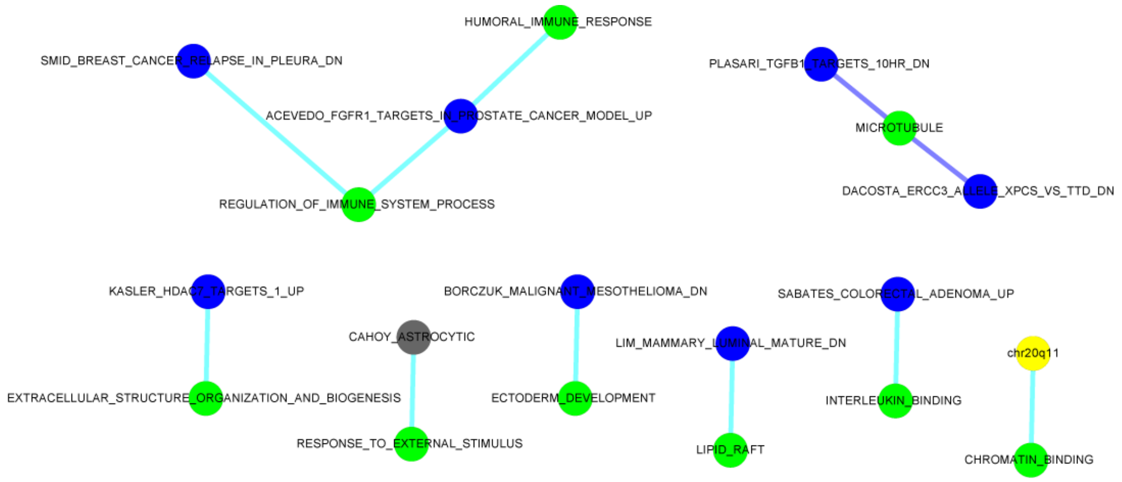

Supplementary Figure S8: The sub-network of gene ontology terms in breast cancer. Extraction of GO terms and their ER-modulated partners from the ER-MGSIN (Fig. 3A of main text).

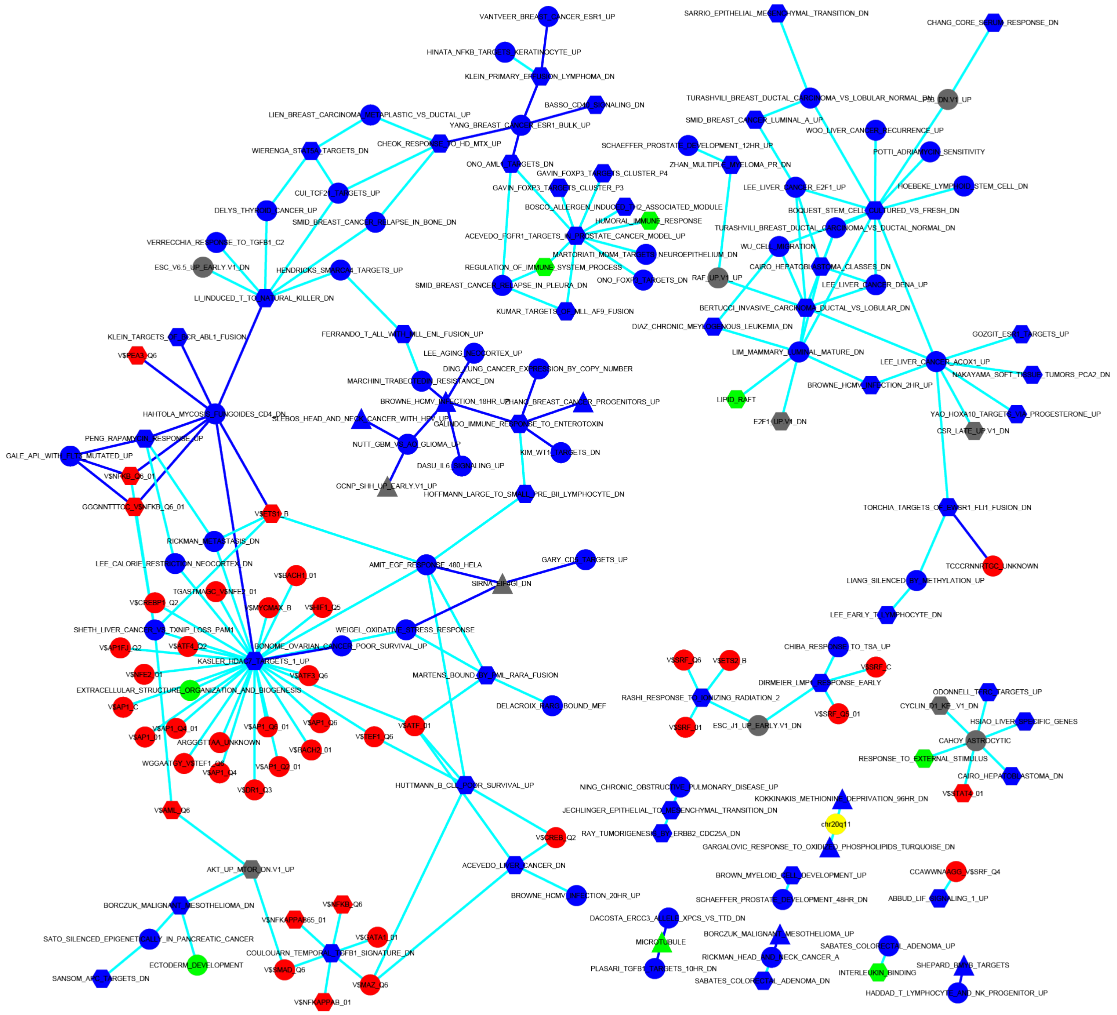

Supplementary Figure S9: The ER dependent prognostic sub-network in breast cancer. Extraction of ER+ dependent prognostic gene sets and their ER-modulated partners from the ER-MGSIN (Fig. 3A of main text), with triangle and hexagon nodes denoting gene sets with ER+ specific positive and negative beta values, respectively. List of gene set interaction pairs is provided in Supplementary Table S4C.

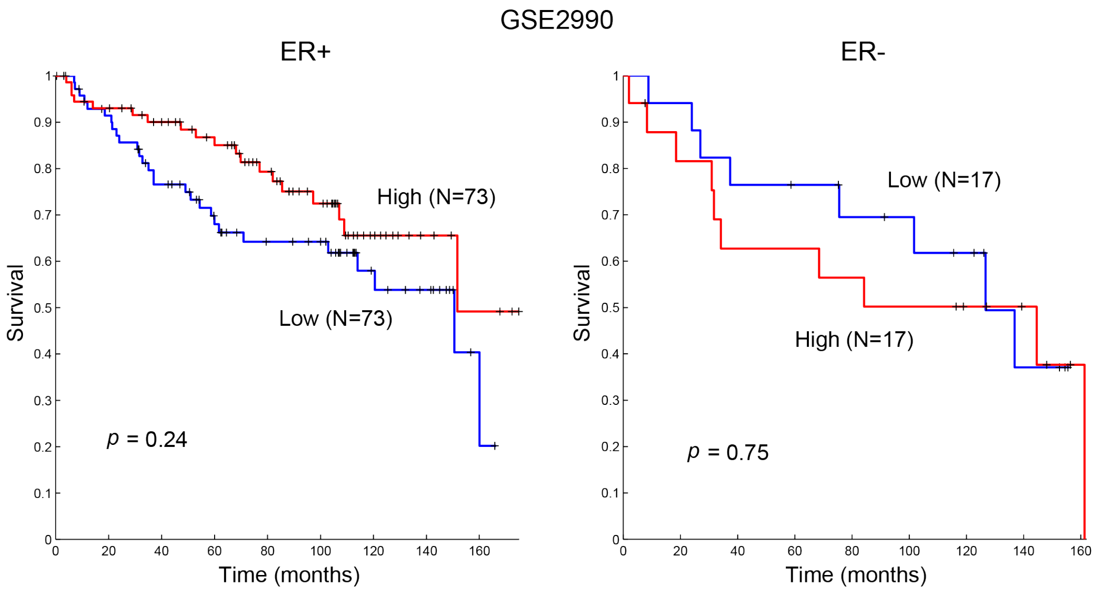

Supplementary Figure S10: Kaplan-Meier curves of COULOUARN_TEMPORAL_TGFB1_SIGNATURE_DN gene set in GSE2990. The TGFβ signature exhibits a trend of ER+ specific association with favorable prognosis, concordant to the results from GSE2034 and GSE4922.

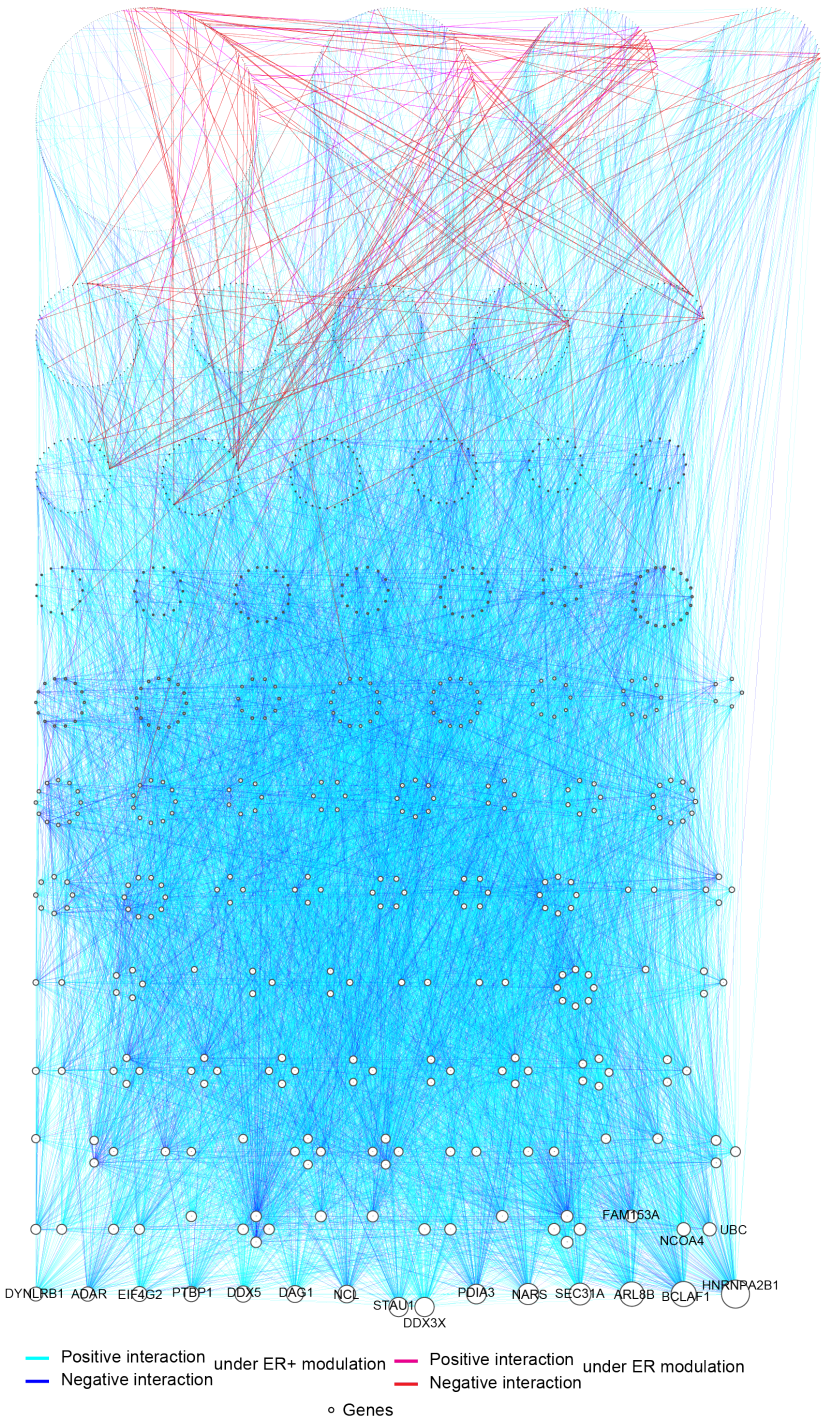

Supplementary Figure S11: The ER-modulated gene interaction network (ER-MGIN) in ovarian cancer. We applied MAGIC to an ovarian cancer dataset and inferred 11,584 significant ER-modulated gene pairs which involved 1,477 genes. Node sizes are proportional to the connectivity of genes, and genes with connectivity >100 are labeled with gene symbols. Genes with identical degree are arranged in one circle. List and summary of ER-MRTPs are provided in Supplementary Table S6A-B.

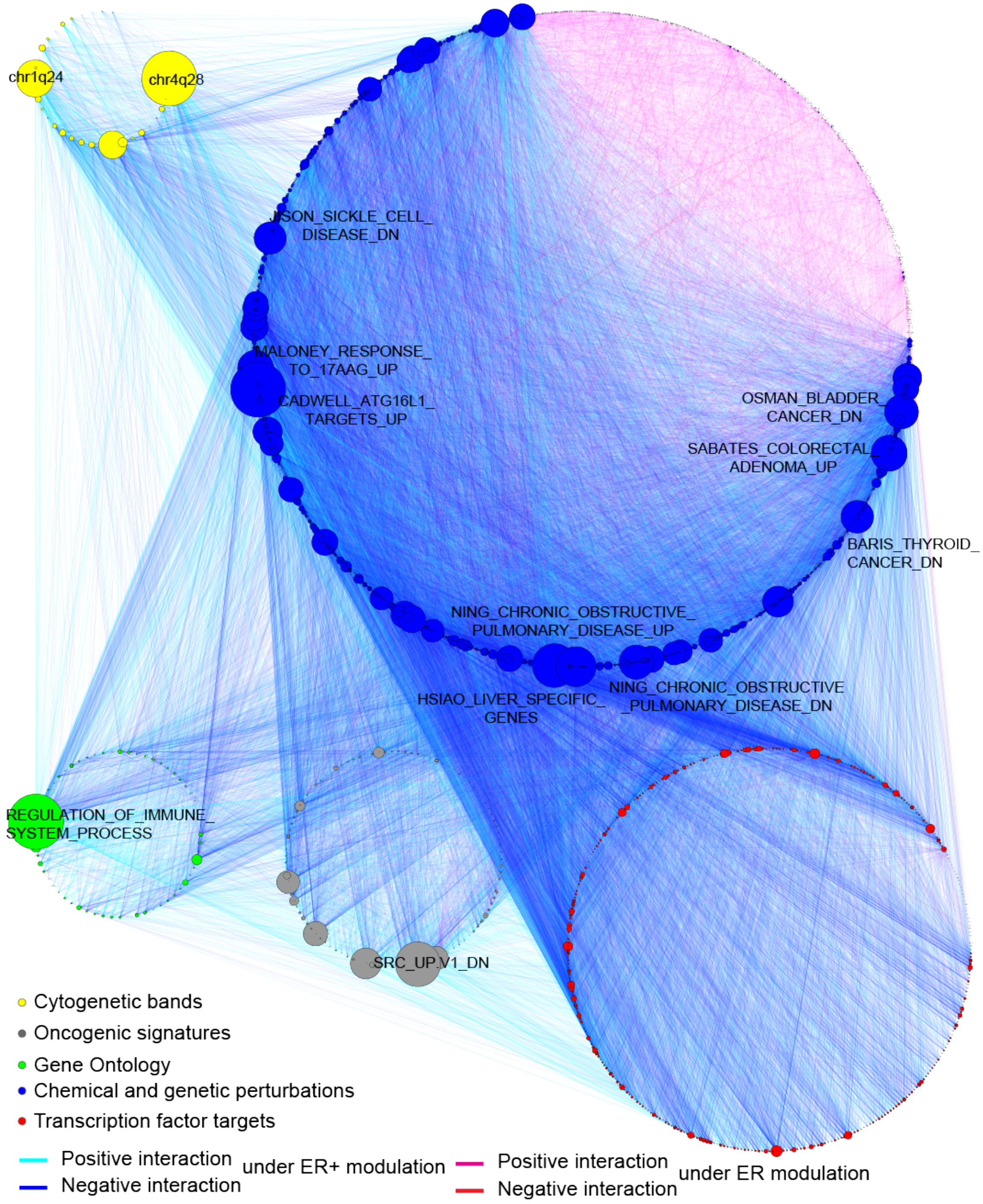

Supplementary Figure S12: The ER-modulated gene set interaction network (ER-MGSIN) in ovarian cancer. We also used MAGIC to analyze the modulated interactions among functions and pathways in ovarian cancer. A total of 38,891 significant ER-modulated gene set pairs which involved 1,517 gene sets were merged into the ER-MGSIN. List and summary of ER-MRTPs are tabulated in Supplementary Table S6C-D.

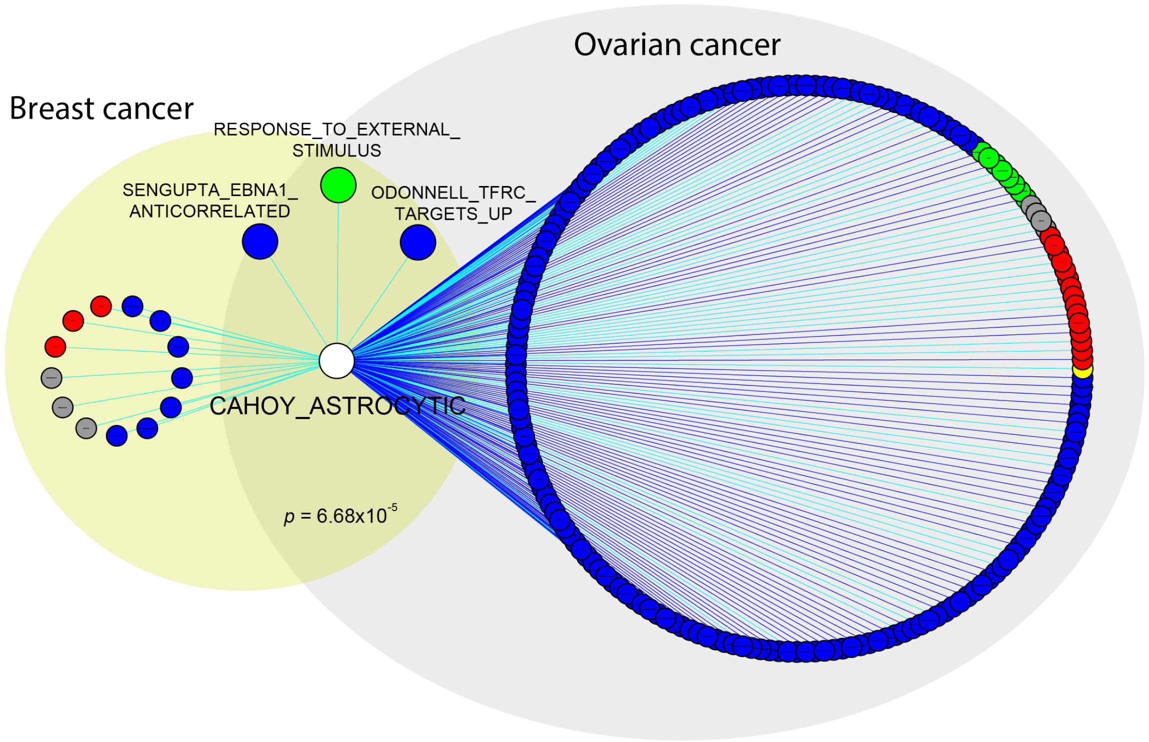

Supplementary Figure S13: The subnetworks of CAHOY_ASTROCYTIC in breast and ovarian cancers. Extraction of CAHOY_ASTROCYTIC and its ER-modulated partners from the ER-MGSINs in breast cancer and ovarian cancer (Fig. 3A of main text and Supplementary Fig. S12). The significance level of overlap between two groups of gene sets was assessed by Fisher's exact test.

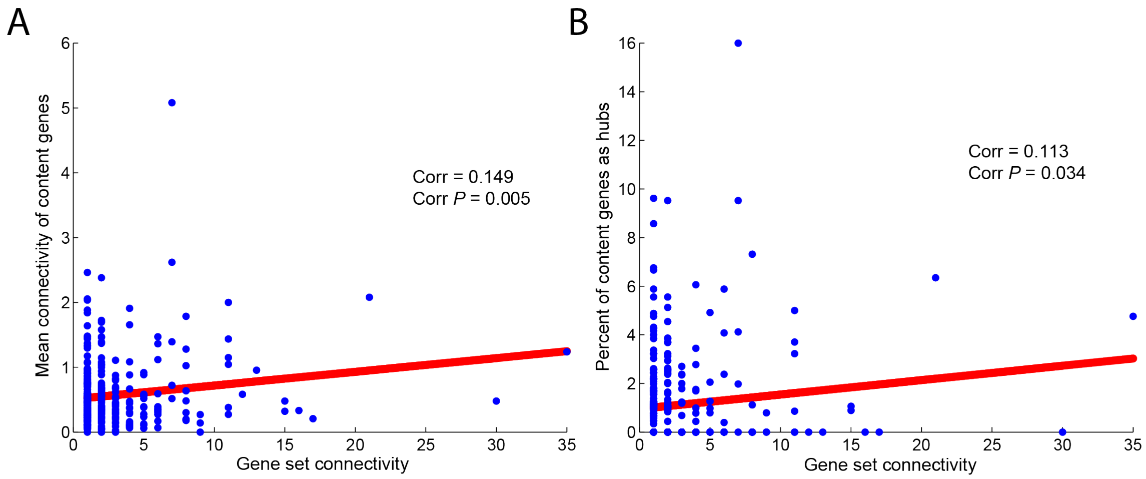

Supplementary Figure S14: Relationship between connectivity of gene sets in ER-MGSIN and the connectivity of their content genes in the ER-MGIN in breast cancer. (A) Scatter plot of gene set connectivity and mean connectivity of genes belonging to the gene set. **(B)** Scatter plot of gene set connectivity and percentage of content genes that appeared as hubs (with connectivity in the top 5%) in the ER-MGIN.

Supplementary Tables

Supplementary Table S1: Summary of microarray datasets used in the study

	GSE2034	GSE2990	GSE4922	GSE26712	TCGA
Usage	Breast cancer discovery	Breast cancer validation	Breast cancer validation	Ovarian cancer discovery	Ovarian cancer validation
Number of ER+/ER− patients (ratio)*	209/77 (2.71)	149/34 (4.38)	211/34 (6.21)	92/92 (1.00)	210/210 (1.00)
Platform	Affymetrix Human Genome U133A Array				Illumina HiSeq 2000 Sequencing
Reference	(Wang, et al., 2005)	(Sotiriou, et al., 2006)	(Ivshina, et al., 2006)	(Bonome, et al., 2008)	(Cancer Genome Atlas Research, 2011)

*Patients with missing estrogen receptor (ER) status were not included for breast cancer datasets.

Supplementary Table S2: Performance of MAGIC in comparison with MI-based methods (balanced design)

Measurement	Method	ρ_{M+}[a]	N=30			N=100			N=300			N=500			N=1000			Mean
			3:1[b]	1:1[b]	1:3[b]	3:1[b]	1:1[b]	1:3[b]	3:1[b]	1:1[b]	1:3[b]	3:1[b]	1:1[b]	1:3[b]	3:1[b]	1:1[b]	1:3[b]	
Precision	MAGIC	0.3	**0.98**	**1.00**	0.67	**1.00**	**0.97**	**0.94**	**1.00**	**1.00**	**0.99**	**1.00**	**1.00**	**1.00**	**1.00**	**1.00**	**1.00**	0.97
		0.7	**1.00**	**1.00**	**0.94**	**1.00**	**1.00**	**1.00**	**1.00**	**1.00**	**1.00**	**1.00**	**1.00**	**1.00**	**1.00**	**1.00**	**1.00**	1.00
		1.0	**1.00**	**1.00**	**1.00**	**1.00**	**1.00**	**1.00**	**1.00**	**1.00**	**1.00**	**1.00**	**1.00**	**1.00**	**1.00**	**1.00**	**1.00**	1.00
	MI	0.3	0.40	0.25	0.33	0.67	0.20	0.50	0.60	0.50	0.67	0.75	0.57	0.50	0.57	0.22	--	0.48
		0.7	0.40	0.36	0.50	0.50	0.50	0.75	**1.00**	0.67	0.33	**0.86**	0.58	0.67	**0.99**	**0.92**	0.57	0.64
		1.0	0.50	0.50	0.70	**1.00**	0.33	0.43	**0.99**	**0.98**	0.50	**1.00**	**1.00**	0.68	**1.00**	**1.00**	**1.00**	0.77
Recall	MAGIC	0.3	0.03	0.01	0.01	0.17	0.07	0.02	0.75	0.48	0.18	**0.96**	**0.82**	0.38	**1.00**	**0.99**	0.80	0.44
		0.7	0.53	0.25	0.07	**1.00**	**0.95**	0.57	**1.00**	**1.00**	**1.00**	**1.00**	**1.00**	**1.00**	**1.00**	**1.00**	**1.00**	0.82
		1.0	**1.00**	**1.00**	**0.85**	**1.00**	**1.00**	**1.00**	**1.00**	**1.00**	**1.00**	**1.00**	**1.00**	**1.00**	**1.00**	**1.00**	**1.00**	0.99
	MI	0.3	0.00	0.00	0.00	0.00	0.00	0.00	0.00	0.00	0.00	0.00	0.00	0.00	0.00	0.00	0.00	0.00
		0.7	0.00	0.00	0.00	0.00	0.00	0.00	0.00	0.00	0.00	0.01	0.00	0.00	0.10	0.01	0.00	0.01
		1.0	0.00	0.00	0.00	0.01	0.00	0.00	0.22	0.05	0.00	**0.89**	0.41	0.01	**1.00**	**1.00**	0.08	0.24
Accuracy	MAGIC	0.3	0.51	0.51	0.50	0.58	0.54	0.51	**0.87**	0.74	0.59	**0.98**	**0.91**	0.69	**1.00**	**1.00**	**0.90**	0.72
		0.7	0.77	0.62	0.53	**1.00**	**0.98**	0.79	**1.00**	**1.00**	**1.00**	**1.00**	**1.00**	**1.00**	**1.00**	**1.00**	**1.00**	0.91
		1.0	**1.00**	**1.00**	**0.92**	**1.00**	**1.00**	**1.00**	**1.00**	**1.00**	**1.00**	**1.00**	**1.00**	**1.00**	**1.00**	**1.00**	**1.00**	0.99
	MI	0.3	0.50	0.50	0.50	0.50	0.50	0.50	0.50	0.50	0.50	0.50	0.50	0.50	0.50	0.50	0.50	0.50
		0.7	0.50	0.50	0.50	0.50	0.50	0.50	0.50	0.50	0.50	0.50	0.50	0.50	0.55	0.51	0.50	0.50
		1.0	0.50	0.50	0.50	0.50	0.50	0.50	0.61	0.52	0.50	**0.95**	0.70	0.50	**1.00**	**1.00**	0.54	0.62
Time (sec.)	MAGIC	0.3	5.3	5.2	5.1	5.2	5.2	5.2	5.2	5.3	5.3	5.3	5.3	5.4	5.5	5.5	5.5	5.3
		0.7	5.1	5.1	5.1	5.2	5.2	5.1	5.3	5.2	5.2	5.3	5.3	5.3	5.5	5.4	5.4	5.2
		1.0	5.1	5.1	5.1	5.1	5.2	5.2	5.7	5.5	5.2	6.1	5.7	8.4	5.8	5.4	5.4	5.6
	MI	0.3	741	726	697	1051	1029	972	1618	1589	1463	2098	2025	1853	3116	3018	2767	1651
		0.7	735	719	697	1030	1020	958	1611	1562	1453	2093	2009	1861	3117	2993	2766	1642
		1.0	731	719	699	1037	1027	960	1613	1588	1607	2664	2590	2225	3193	2968	2741	1757

Measurement numbers greater than 0.80 are labeled in bold.

[a]Correlation coefficient in M+ samples for M-modulated pairs

[b]Ratio between numbers of M+ and M− samples

Supplementary Table S5: Validation of ER modulated interaction between COULOUARN_TEMPORAL_TGFB1_SIGNATURE_DN and three NFκB target gene sets in breast cancer

	GSE2990				GSE4922			
	Corr. in ER+	Corr. in ER−	ΔI^{adj}	*P*-value	Corr. in ER+	Corr. in ER−	ΔI^{adj}	*P*-value
V$NFKAPPAB65_01	0.41	-0.14	0.42	1.67e-6	0.54	0.10	0.61	~0
V$NFKAPPAB_01	0.43	-0.14	0.45	2.08e-7	0.53	0.01	0.65	1.11e-16
V$NFKB_Q6	0.39	-0.10	0.43	1.53e-6	0.53	0.03	0.64	1.11e-16

Supplementary Table S7: Validation of ER modulated interaction between COULOUARN_TEMPORAL_TGFB1_SIGNATURE_DN and three NFκB target gene sets in ovarian cancer

	GSE26712			
	Corr. in high-*ESR1*	Corr. in low-*ESR1*	ΔI^{adj}	*P*-value
V$NFKAPPAB65_01	0.60	0.51	0.10	0.06
V$NFKAPPAB_01	0.57	0.40	0.17	0.008
V$NFKB_Q6	0.58	0.46	0.12	0.03
